# Combined benznidazole and pentoxifylline therapy improves behavioral and cognitive changes in association with the regulation of systemic inflammatory profile in chronic experimental Chagas disease

**DOI:** 10.1371/journal.pone.0334708

**Published:** 2025-11-14

**Authors:** Glaucia Vilar-Pereira, Leda Margarita Castaño-Barrios, Isabela Resende Pereira, Ana Paula da Silva Pinheiro, Thayse do Espírito Santo Protásio da Silva, Lina L. Hernandez-Velasco, Priscila Silva Grijó Farani, Aditi Kulkarni, Sourav Roy, Hílton Antônio Mata dos Santos, Raquel de Oliveira Lopes, Luzineide Wanderley Tinoco, Constança Britto, Otacílio Cruz Moreira, Andrea Alice Silva, Joseli Lannes-Vieira

**Affiliations:** 1 Laboratório de Biologia das Interações, Instituto Oswaldo Cruz/Fiocruz, Rio de Janeiro, Rio de Janeiro, Brazil; 2 Departamento de Microbiologia e Parasitologia, Instituto de Biologia, Universidade Federal de Pelotas, Pelotas, Rio Grande do Sul, Brazil; 3 Departamento de Matematicas, Pontificia Universidad Javeriana, Bogotá, Colombia; 4 Laboratório de Virologia e Parasitologia Molecular, IOC/Fiocruz, Rio de Janeiro, Rio de Janeiro, Brazil; 5 Department of Biological Sciences and Border Biomedical Research Center, University of Texas at El Paso, El Paso, Texas, United States of America; 6 Escola de Farmácia, Universidade Federal do Rio de Janeiro, Rio de Janeiro, Rio de Janeiro, Brazil; 7 Laboratório de Análise e Desenvolvimento de Inibidores Enzimáticos, Universidade Federal do Rio de Janeiro, Rio de Janeiro, Rio de Janeiro, Brazil; 8 Programa de Pós-Graduação em Farmacologia e Química Medicinal, Instituto de Ciências Biomédicas, Universidade Federal do Rio de Janeiro, Rio de Janeiro, Rio de Janeiro, Brazil; 9 Laboratório Multiusuário de Análises por Ressonância Magnética Nuclear (LAMAR), Instituto de Pesquisas de Produtos Naturais (IPPN), Universidade Federal do Rio de Janeiro, Rio de Janeiro, Rio de Janeiro, Brazil; 10 Laboratório de Biologia Molecular e Doenças Endêmicas, IOC/Fiocruz, Rio de Janeiro, Rio de Janeiro, Brazil; 11 Laboratório Multidisciplinar de Apoio à Pesquisa em Nefrologia e Ciências Médicas, Faculdade de Medicina, Universidade Federal Fluminense, Niterói, Rio de Janeiro, Brazil; Oswaldo Cruz Foundation: Fundacao Oswaldo Cruz, BRAZIL

## Abstract

Chronically *Trypanosoma cruzi*-infected mice show signs of behavioral and cognitive changes, resembling aspects of Chagas disease patients. Inflammatory mediators, such as cytokines and nitric oxide (NO) have been linked to mental disorders. Preclinical studies showed the partial effects of the trypanossomicidal drug benznidazole (Bz) on mnemonic alterations. Here, we investigated the participation of the parasite and systemic inflammatory profile in behavioral and cognitive changes, using Bz combined with the immunoregulator pentoxifylline (PTX). Chronically *T. cruzi*-infected C57BL/6 mice were treated with Bz (25 mg/Kg/day) and PTX (20 mg/Kg/day) as mono or combined therapies, submitted to behavioral tests, and canonical biological stressors were analyzed. Bz therapy had no effects on anxiety, but partially ameliorated innate compulsive behavior, depression, and memory loss, while PTX and, mainly, Bz + PTX had a broader beneficial effect on these changes. Bz and Bz + PTX reduced parasitemia. The three therapies decreased the parasite burden in the brain. Bz and Bz + PTX therapies reduced oxidative stress in the brain tissue, while PTX and Bz + PTX therapies efficiently controlled the elevated concentrations of GABA/glutamate in the cerebral cortex. Even after parasite control, serum concentrations of NO and tumor necrosis factor (TNF) enhanced as the disease progressed. Bz and, mainly, Bz + PTX treatments reduced NO levels. The three therapeutic schemes hamper the progressive increase of TNF levels. Reanalysis of available data on the systemic miRNA transcriptome supports the beneficial role of Bz + PTX therapy on pivotal hubs involved in inflammation of the central nervous system and neurodegenerative disorders. Moreover, principal components analysis (PCA-2D and 3D projections) underlined the distinction between the noninfected and vehicle-treated infected groups, while Bz + PTX-treated infected mice were closer to noninfected controls. The combined Bz + PTX therapy reduced parasite load and regulated pivotal neurochemical changes in the brain and the systemic inflammatory profile, improving behavioral and cognitive changes in a model of Chagas disease.

## Introduction

Chagas disease (CD), a neglected tropical disease caused by the protozoan parasite *Trypanosoma cruzi*, afflicts 6–7 million people. In comparison, 75 million people are at risk of infection in endemic regions in Latin America [1]. Due to migrations, CD has become a global health concern in non-endemic countries in North America, Europe, and Oceania [2]. Behavioral and cognitive disorders have been described in chronic infectious diseases caused by viruses, bacteria, and parasites [3]. CD patients may be affected by headache, confusion, and speech disorders [4], sleep dysfunction [5], anxiety and depression [6,7], attention deficits and memory disorders [5,8,9], which may contribute to their poor quality of life [8]. In elderly CD patients, cognitive abnormalities have been associated with positive serology, independent of electrocardiographic changes [10]. Further, in 37% of a group of CD patients, depression was independent of cardiac dysfunction [7]. Poverty-associated social and psychological stressors may contribute to behavioral disorders [11]. These are potential determinants of the mental disorders afflicting CD patients, mostly exposed to poverty, discrimination, and stigmatization [12]. Preclinical studies support that infection-triggered biological and molecular stressors may also contribute to behavioral and cognitive changes in CD. Sleep alterations were detected in *T. cruzi*-infected rats [13]. In chronically *T. cruzi*-infected mice, anxiety and depression, independently of sickness-like behavior [14,15], and memory deficits [16,17] were shown. In C57BL/6 infected with the Colombian *T. cruzi* strain the onset of behavioral and cognitive changes is sequential, initiating with the loss of the innate compulsive behavior, followed by anxiety and depressive-like behavior, ending with progressive (object recognition, spatial and aversive) memory impairments [17,18]. However, the biological and molecular stressors underpinning these changes in *T. cruzi* infection remain to be fully comprehended. Moreover, therapeutic strategies that can improve the prognosis for these abnormalities are a current challenge.

High levels of the neurotransmitters gamma-aminobutyric acid (GABA) and glutamate disrupt brain physiology, sustaining depression [19]. Further, a reduction in expression of the neurotrophin brain-derived neurotrophic factor (BDNF) may lead to mental disorders with anxiety [20] and a decline in learning and memory formation [21]. In experimental chronic *T. cruzi* infection, the antidepressant fluoxetine, a selective serotonin reuptake inhibitor reversed depression, as expected, but also ameliorated cognitive changes, favoring a more balanced profile of GABA and glutamate, and improving BDNF expression [18]. Memory impairments were also related to *T. cruzi* parasite persistence and oxidative stress in the brain tissue, biological processes partially reversed by the antiparasitic drug benznidazole (Bz) [17]. Thus, in chronically *T. cruzi*-infected mice, behavioral and cognitive changes were ameliorated, but not resolved, by targeting a neurotransmitter pathway with fluoxetine or parasite load with Bz, implying that these clinical changes may be underpinned by a more complex network of biological stressors.

An increase in lipid peroxidation, a marker of oxidative stress, and central nervous system (CNS) inflammation, as well as systemic inflammatory processes, are proposed to underly neurodegenerative diseases involving memory deficits such as Alzheimer disease [22,23]. Further, nitric oxide (NO), as a neuromediator, plays an important physiological role in learning and memory formation [24]. High NO levels may represent a link between inflammation and oxidative stress, having neurotoxic effects in chronic disorders such as Alzheimer disease [25]. CD pathogenesis is proposed to rely on a parasite-driven systemic inflammatory profile, which may reverberate in target tissues, contributing to intrinsic alterations and aggravating clinical outcomes such as cardiopathy [26,27] and behavioral and cognitive changes [3]. Systemic inflammatory profile, enriched in NO and cytokines such as tumor necrosis factor (TNF) and interferon-gamma (IFNγ), is detected in CD patients [28–32] and in experimental chronic *T. cruzi* infection [33–37] related with the severity of cardiac disease. In experimental CD, the potential role of inflammatory mediators in CNS commitment is supported by *in vitro* data showing that IFNγ and TNF fuel the infection of astrocytes by *T. cruzi* through a complex network involving serotonin and creating a NO- and glutamate-enriched milieu [18]. These findings may ultimately help explain the behavioral changes in infected mice, thus opening opportunities to test therapeutic immunoregulatory interventions.

Given the lack of specific therapeutic tools, CD is treated like other neglected diseases, using medications to mitigate symptoms [26,27]. The methylxanthine pentoxifylline (PTX), a phosphodiesterase inhibitor with pleiotropic functions, shows therapeutic potential as an anti-inflammatory agent for cardiac diseases [38] and SARS-Cov2 mimicking respiratory disorder [39]. Also, PTX and other methylxanthines have been proposed to treat neurodegenerative diseases due to their antioxidative properties [40,41]. Further, PTX has been used as an adjuvant tool to treat leishmaniasis, a parasitic disease with intense systemic inflammatory processes [42]. Moreover, in chronically *T. cruzi*-infected mice, PTX therapy reduced signs of depressive-like behavior [14], regulated abnormal T-cell activation, and improved heart dysfunction [36,37]. Altogether, these findings let us explore the contribution of the parasite and systemic inflammatory profile as biological stressors underpinning behavioral and cognitive abnormalities in chronic *T. cruzi* infection. To test this idea, C57BL/6 mice were infected with the Colombian *T. cruzi* strain, the antiparasitic drug Bz and the immunoregulator PTX were administered as mono or combined therapies, for 30 consecutive days, starting at 120 days post-infection (dpi), when most behavioral and cognitive alterations are already installed [17,18]. After therapies, behavioral and cognitive changes were evaluated, and canonical biological stressors in the CNS (GABA, glutamate, BDNF, lipid peroxidation) and systemic inflammatory molecules (NO, TNF) were analyzed. Based on the data obtained, we reanalyzed our data on mRNA expression focused on immune response-related pathways [43] and the transcriptome profile of small non-coding microRNA (miRNA) in heart tissue [44], as readouts of the systemic effects of Bz-based therapies exploring CNS-related pathways. Further, we performed a multiparameter study to establish a correlation between behavioral, neurochemical, and immunological changes. Lastly, we used principal components analysis (PCA-2D and 3D projections) to identify and visualize the similarities between groups that received the therapeutic schemes.

## Materials and methods

### Ethics statement

All experimental procedures were approved by the Animal Ethics Committee of Oswaldo Cruz Institute/Fiocruz (licenses L-006/2018, L-002/2023-A2). The *in vivo* data presented was obtained from two independent experiments (Register Books #49, #53, #66, #71, and #75, LBI/IOC-Fiocruz) conducted according to the Brazilian Federal Law 11.794 (October 8^th^, 2008). The drawings were made by hand using licensed PowerPoint and Adobe Photoshop 2024.

### Experimental design

The Author´s Checklist (S1 Checklist) describes the experimental checklist. The Institute of Science and Technology in Biomodels (ICTB) of the Oswaldo Cruz Foundation (Fiocruz) provided C57BL/6 strain (H-2^b^) 5–7-week-old female mice (107 mice in total). After arrival, mice were numbered and grouped into 3–5 mice per polypropylene cage containing Pinus sawdust. Cages were numbered and randomly sorted for the experimental infection and analysis at the indicated time points. Cages were kept in microisolators, and mice received water and grain-based chew food *ad libitum*. Mice were adapted for 14 days in a plastic igloo-enriched cage in specific pathogen-free conditions, with light and noise control to minimize stress. After this period, mice were infected and analyzed as described in the Workflow chart (S1 Fig), according to The Arrive guidelines 2.0 [45]. GVP, IRP, AAS and JLV performed two independent experiments. Groups were composed as follows: **Kinetic** and **Pre-therapy**: non-infected (NI) controls (10 mice = 5 + 5); *T. cruzi*-infected (18 mice = 9 + 9), analyzed from 14 to 150 days post-infection (dpi) for NO and TNF serum levels. **Therapy groups** (therapy 120–150 dpi; analysis at 149–151 dpi – referred to as “150 dpi”): non-infected (NI) controls (10 mice = 5 + 5); and *T. cruzi*-infected groups: Vehicle-treated (Veh; 19 mice = 9 + 10); pentoxifylline-treated (PTX; 15 mice = 7 + 8), benznidazole-treated (Bz; 18 mice = 8 + 10), Bz + PTX-treated (Bz + PTX; 17 mice = 7 + 10). All infected mice were evaluated for parasitemia in the “Kinetic”, “Pre-therapy” and “Therapy” experiments (S1 Fig).

### Infection by *Trypanosoma cruzi*, parasitemia and clinical follow-up

Mice were infected intraperitoneally with 100 blood trypomastigote (bt) forms of the Colombian *T. cruzi* strain (DTU-TcI) suspended in 0.2 mL of sterile saline buffer, and parasitemia was performed weekly, as previously described [33]. Weekly, death was recorded, and clinical signs were analyzed (piloerection, apathy, prostration, mobility, posture, aggressive behavior, pain). Body weight loss (which may reveal loss of appetite, diarrhea, and absorption disturbance) was assessed using a rodent weighing scale (Sartorius scale, ED623S-OCE, USA). Exclusion criteria were negative parasitemia (35–45 dpi), any death outside of planned euthanasia and according to pre-established endpoints conditions such as body weight loss (≥ 30% of the initial weight), injuries from fights, pain, posture, ataxia, and immobility. Parasitemia was evaluated microscopically in 5 µL of tail vein blood according to Pizzi-Brener method [46]. At 152 dpi, mice were euthanized under anesthesia, and tissues (encephala and hearts) were collected in RNA later stabilization solution (Invitrogen, USA) for processing prior to molecular assays. Heart tissues were processed for mRNA and miRNA expressions, and the data were previously published [43,44].

### Therapy administration

After 120 dpi, when mice show clinical signs of behavioral and cognitive changes [17,18], we started the therapeutic intraperitoneal injection with apyrogenic saline (BioManguinhos/Fiocruz, Brazil) containing PTX (20 mg/Kg/day; Trental, Sanofi-Aventis) and/or Bz (25 mg/Kg/day; LAFEPE) by gavage using apyrogenic water (BioManguinhos/Fiocruz, Brazil), for 30–31 consecutive days [37,43,44]. Non-infected (NI group) and infected mice (Veh group) received an intraperitoneal injection with apyrogenic saline and gavage with apyrogenic water as control groups and groups receiving monotherapies (Bz or PTX), also received complementary Veh treatment, to rationally submit all mice to the stress of daily manipulation as were mice treated with Bz + PTX.

### Behavioral tests

Our group established the environmental conditions and behavioral tests according to the literature and fully described them in our previous publications [15,17,18,47]. Briefly, behavioral tests were conducted between 8:00 a.m. and 3:00 p.m. and recorded using a DSC-DVD810 video camera (Sony, USA). The experiments were conducted in a controlled environment with 12 hours of light and 12 hours of dark at a stable room temperature of 22 ± 2 °C and an air conditioner noise level of approximately 40 dB. This setting was designed to reduce stress and enhance the mice’s familiarity with the environment. Mice were submitted to behavioral tests at 149–151 dpi, thereafter, stated as “150 dpi” in “Therapy experiments”. They were not retested, but all were reused in different tests to reduce the number of animals used. Behavioral tests were performed from less stressful tests to the more stressful: spatial habituation memory test (OFT2), novel object recognition memory test (NORT), elevated plus maze test (EPMT), marble burying test (MBT), tail suspension test (TST), and aversive shock evoked test (ASET). The open field test (OFT) is based on mice’s tendency to explore new environments. To evaluate the habituation spatial memory (OFT2), on the first day (day 1, training session), mice are placed in the upper corner of the testing area and allowed to explore for 5 minutes. The long-term memory test (day 2) was performed 24 hours after the training. In this test, the procedure was repeated, the 5-minute session was recorded, and memory retention was evaluated by counting the number of total lines crossed during the test session. Individual baseline differences were corrected using the change ratio score to compare behavior during the training (day 1) and test session (day 2). The data are shown as discrimination index (DI), as follows: DI = number of crossed lines day 2/ (number of crossed lines day 1 + number of crossed lines day 2). The NORT is based on the innate preference of NI normal rodents to explore a novel object rather than a familiar one. The test was initiated two hours after the OFT2. The mouse was allowed to explore two similar objects for five minutes. After 24 hours (day 3), one object was replaced (a novel object), and mice were submitted to a five-minute session [48]. According to a previous publication [17], the DI for NORT was calculated as follows: DI = time exploring the novel object/ (time exploring the novel object + time exploring the familiar object).

Anxiety-like behavior was assessed using EPMT, also based on the natural tendency of mice to explore a new environment. The behavioral parameters related to anxiety were the (i) number of entries (EPMT-entries) and (ii) time spent (EPMT-time) in the open arms. An entry was scored when all four mouse paws were placed on an arm [49]. Mice exhibit repetitive compulsory burying behavior in the presence of aversive stimuli; this is the basis of the MBT. Here, glass beads were used as aversive stimuli, and the number of buried glass beads in sawdust was registered at 5, 10, 15, 20, 25, and 30 minutes, as described [16].

The immobile posture or apathetic behavior in response to short-term moderately stressful or uncomfortable inescapable conditions is interpreted as depressive-like behavior in TST [50]. TST was performed using the tail suspension apparatus (TST, Insight, Brazil), as previously described [47]. Data are expressed as immobility time in seconds (s). As we previously described [17], the aversive shock-evoked test was performed using the inhibitory avoidance apparatus (EP 104MR, Insight, Brazil). In a pre-exposure session, mice were placed on the platform and allowed to explore the box freely for 5 minutes without foot shock. Two hours later, a training session was carried out when mice were placed on the platform, and an automatic device measured their latency to step down on the grid with all four paws. Immediately after stepping down on the grid, the animals received a 3.0-sec scrambled foot shock (0.6 mA). One to five stimulations were required for memory acquisition, and the latency between stimuli was registered (training session, ASET1). In the test session, 24 hours after training, no foot shock was administered, and the step-down latency (maximum 120 seconds) was used to measure memory retention (test session, ASET2). Data are expressed as latency measured in seconds (s), as described [51].

### Blood obtention, euthanasia and encephalon tissue obtention

After topical eye drops anesthesia, mice were bled by orbital plexus, and serum was stored at −80 °C. Mice were euthanized at the endpoints (120 dpi and 150 dpi) using CO_2_ inhalation in an appropriate chamber, allowing 70% CO_2_ saturation for 2–3 minutes, followed by decapitation. Encephala and hearts were collected and weighed. Considering the importance of the cerebral cortex and hippocampus for behavioral and cognitive changes, these areas were dissected. Briefly, after the brain was removed and weighed, it was placed in an ice-cold saline solution to preserve tissue integrity [52]. The cortex and hippocampus were identified using anatomical landmarks [53]. Each region was carefully dissected using fine surgical instruments, ensuring that the boundaries between regions were respected to avoid contamination. The cortex was separated from the hippocampus by making precise cuts along the rhinal and the hippocampal fissures. After dissection, each brain region was preserved in RNA later (AM7021, Thermo Fisher Scientific, USA). The tissues were immediately stored at −80 °C for the following assays: BDNF; lipid peroxidation by evaluation of tissue levels of thiobarbituric acid reactive species (TBARS); GABA/glutamine determination by nuclear magnetic resonance; and parasite load determination by quantitative PCR (qPCR).

### Determination of parasite load in the CNS by quantitative PCR (qPCR)

Mice from two independent experiments were euthanized, and the encephalon was removed and dissected [52]. DNA was extracted from dissected cerebral cortex and hippocampus samples, and the parasite load was measured by qPCR [17]. The standard curve was prepared by spiking 10–100 mg of non-infected brain tissue with 10^5^ *T. cruzi* trypomastigotes (Colombian strain). DNA was extracted and serially diluted (1:10) in TE buffer. This standard curve was subjected to qPCR assays for the *T. cruzi* satDNA and mouse GAPDH targets. The number of parasite equivalents and the tissue mass (in milligrams) of the samples were calculated by absolute quantification, using the respective standard curves (S2 Fig). Finally, the parasite load was calculated by dividing the number of parasite equivalents by the tissue mass (mg), using the formula:


$$\text{Parasite Load}=\frac{\text{Parasite equivalents}}{\text{Tissue mass }(\text{mg})}$$

### BDNF gene expression analysis by real-time quantitative RT-PCR

For BDNF studies, the cerebral cortex was removed and processed as previously described [18]. Total RNA was extracted from the same sample used to quantify the parasite load using TRI-Reagent (Sigma-Aldrich, St. Louis, MO, USA). The procedures to obtain RNA, cDNA, and gene expression analysis by RT-qPCR using the TaqMan Assays (Applied Biosystems, USA) were described elsewhere [18]. Assays were performed according to the manufacturer’s instructions for BDNF neurotrophic factor (Mm04230607-s1) as the target gene and GAPDH (Glyceraldehyde-3-phosphate dehydrogenase; Mm99999915g1) and HPRT (Hypoxanthine-guanine phosphoribosyltransferase; Mm01545399m1) as reference genes, using Viia7 (Applied Biosystems, USA), as previously described [18].

### Determination of glutamate and GABA neurotransmitters in the CNS by nuclear magnetic resonance

The cortex and hippocampus were analyzed for GABA and glutamate neurotransmitters using nuclear magnetic resonance. All procedures to obtain samples were previously described [18]. Proton Nuclear Magnetic Resonance (^1^H NMR) spectra were acquired at 25 °C on a VNMRS-500 (Agilent, USA) spectrometer at 499.78 MHz. Spectra were acquired with a spectral width of 7022 Hz, with 16384 points and 64 accumulations. For quantitative analysis, a relaxation interval (d1) of 35 s was used. Nuclear magnetic resonance spectra were processed with the MestReNova v10.0.1-14719 program.

### Lipid peroxidation evaluation in the CNS

Mice were euthanized, encephala removed, the cortex and hippocampus dissected [52], and homogenates prepared. Malondialdehyde and other TBARS, products of degradation of hydroperoxides and lipid peroxides formed during the oxidation of fatty acids were determined through their reaction with thiobarbituric acid (TBA) under heat and acidic pH, forming a pink-colored complex, as previously described [17]. Ten percent (w/v) of tissue homogenate was mixed with 8.1% sodium dodecyl sulfate (SDS), 20% acetic acid pH 3.5, and 0.8% TBA and incubated at 95 °C for 1 hour. After incubation, the reaction product was extracted with n-butanol (1:1), centrifuged for 5 minutes at 800 x g, and supernatants were collected. This MDA-TBA2 adduct exhibits a conjugated double-bond system, which absorbs visible light with a peak at 532 nm, read using a spectrophotometer (Spectra Max M5, Molecular Devices, USA).

### NO quantification in sera

Nitrate and nitrite (NO_x_) were determined in serum samples after deproteinization and reduction of nitrate by vanadium (III) chloride combined with detection by the acidic Griess reaction, and using a standard curve of 0.8 to 100 μM NaNO_2_ and NaNO_3_, as described elsewhere [54].

### Determination of cytokines in sera

The BD Cytometric Bead Array (CBA) Mouse Inflammation kit (catalog 552364, BD Bioscience) measured serum IL-6, IL-10, MCP-1/CCL2, IFN-γ, TNF, and IL-12p70 protein concentrations using standard calibration curves according to the manufacturer’s recommendations. The samples were analyzed using the 13-Color CytoFLEX-S flow cytometer (BeckmanCoulter, USA). Cytokine concentrations were determined using reference curves and expressed in pg/ mL using the FCAP Array Software.

### Analysis of mRNA and miRNA in cardiac tissue, pathway enrichment, and miRNA target filter analysis

This study builds upon previous findings highlighting the absence of cytokine gene expression within the CNS but elevated cytokine levels in serum of chronically *T. cruzi*-infected C57BL/6 mice [18]. Our study aimed to delineate the systemic inflammatory profile through a detailed examination of TNF expression in cardiac tissues assessed as previously described [37], complemented by mRNA data [43]. This investigation utilized TaqMan probes targeting 92 immune response genes alongside four endogenous reference genes for normalization and quantification. Such an approach allowed for the establishment of a correlation between specific gene expressions in cardiac tissues (including TNF, IFNγ, IL-10, IL-12a, IL-12b, IL-2, IL-6) and serum inflammatory mediators (NO, TNF, IL-6), thus elucidating the ramifications of *T. cruzi* infection and Bz-based treatments on these markers. Data normalization was conducted using NI controls.

Considering the limitation in accessing miRNA transcriptome data within brain tissues and serum samples, an exploratory approach was adopted to investigate the systemic miRNA profile under *T. cruzi* infection and therapeutic protocols. The aim was to identify potential targets for differentially expressed miRNAs (DEMs) under various conditions of infection and treatment, drawing upon miRNA datasets provided by Farani et al. [44], that utilized pre-printed TLDA 384 wells microfluidic cards (TaqMan™ Array Rodent MicroRNA A + B Cards Set v3.0, Applied Biosystems, ThermoFisher Scientific, USA/Cat no. 4444909) that contained FAM/NFQ-MGB labelled probes specific to 752 mature miRNAs and four endogenous small nucleolar RNAs candidates for data normalization and relative quantification. The Ingenuity Pathway Analysis (IPA; Qiagen, Redwood City, CA) software was pivotal for this analysis. It facilitated the exploration of direct and indirect relationships between miRNAs and their potential target genes within CNS-related pathways and evaluated the systemic effects of Bz and Bz + PTX therapies. DEMs were mapped to the IPA knowledgebase via miRNA IDs, and a miRNA target filter analysis was employed to pinpoint both experimentally observed targets and those predicted with high confidence. A core analysis was then conducted with the identified targets to discern canonical pathways, predominant diseases, and affected functions. Using the “Grow” and “Connect” tools within IPA allowed for a visualization of the intricate interactions between miRNAs and mRNAs, alongside additional direct and indirect connections amongst molecules at a pre-determined confidence level. This comprehensive analytical framework, enhanced through the “Path Designer” tool for clear visualization, facilitated an in-depth understanding of the molecular interactions at play, offering novel insights into the systemic inflammatory response and therapeutic avenues in this preclinical model of CD.

### Statistical analysis

The sample size was determined based on the experience of our group and previous studies using the model of experimental chronic chagasic cardiomyopathy [33,34,37] and behavioral alterations [15,17,18]; therefore, no formal sample size was calculated. The Shapiro-Wilk test was used to assess the normality of the data. For normally distributed data formed by two groups, differences were analyzed with Student’s t-test. For normally distributed data of more than two groups, the difference between groups was analyzed using the parametric one-way ANOVA test, corrected with Tukey post hoc test with multiple comparisons, with a 95% confidence. Non-normally distributed data were analyzed using the non-parametric Mann-Whitney test for groups with two groups or Kruskal-Wallis on ranks for analysis with more than two groups, followed by the post-hoc Dunn’s multiple comparisons tests. Data from two independent experiments were used. All statistical tests were performed with GraphPad Prism 8.0.1 (La Jolla, CA, USA). Data are expressed as mean ± SD. Differences were considered statistically significant when *p < *0.05.

To visualize simultaneously the most important variables involved in our analysis, we computed principal-component analysis (PCA), which allowed us to identify graphically the analyzed parameter and recognize the variables associated with the performance of the NI controls and the Veh-, Bz-, PTX-, Bz + PTX-treated *T. cruzi*-infected groups. To enhance the PCA, we only included variables that showed significant correlation based on the Spearman correlation significant test (p < 0.05). All analyzed parameters of four mice per group were included in PCA analyses. PCA was performed on scaled normalized expression values using the built-in R function PCA from package FactoMineR (v1.34), and the plots were generated using the ggplot2 (v3.3.3), factoextra (v1.0.7) and pca3d (v0.10.2) R packages. All R analysis was carried out using the RStudio environment (v1.4.1103) [55].

## Results

### Effects of PTX and Bz therapies on parasitemia and parasitism control in chronically *T. cruzi*-infected mice

**Fig 1:**
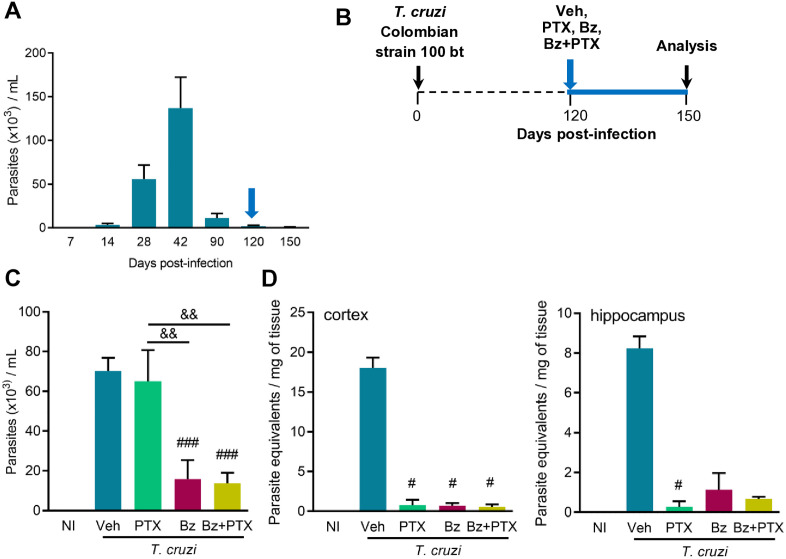
Effects of PTX and Bz therapies on parasitemia and brain parasitism in chronically infected mice. (A) The graph shows the kinetic of parasitemia (7-150 dpi). The blue arrow indicates the beginning of therapies (120 dpi). (B) The scheme shows that mice infected with 100 bt forms of the Colombian strain (*T. cruzi*) were treated with Veh, PTX, Bz, or Bz + PTX, daily from 120-150 dpi. (C) The graph shows parasitemia in Veh-, PTX-, Bz-, and Bz + PTX-treated mice at 150 dpi. (D) The graph shows parasite DNA quantified by qPCR in the cerebral cortex and hippocampus of non-infected control (NI) and *T. cruzi*-infected mice at 150 dpi. Representative data of two independent experiments. Color code: Grey bars indicate NI, blue bars show Veh-treated, green bars indicate PTX-treated, pink bars show Bz-treated, and yellow bars indicate Bz + PTX-treated infected mice. Each bar shows mean ± SD. ^###^, *p* < 0.001, comparing treated groups with Veh-treated *T. cruzi*-infected mice; ^&&^, *p* < 0.01, comparing Bz- and Bz + PTX-treated grou*p*s with PTX-treated infected mice.

Experiments were performed according to the experimental Workflow (S1 Fig). S1 Table shows data used to build graphs and figures. During treatment all mice survived. At 120 dpi, non-infected and *T. cruzi* groups had similar body weight (S3A Fig). At 150 dpi, mice groups treated with Bz (*p* = 0.083) and Bz + PTX (*p* = 0.023) showed reduced body weight compared with age-matched non-infected mice (S3A Fig). Crucially, in Bz-treated groups we noticed a significant reduction of infection-associated splenomegaly (S3B Fig), a highly deleterious aspect of chronic Chagas infection, associated with increased cell activation [33]. No signs of injury, ataxia, or pain were registered. A parasitemia peak was detected at 42 dpi, parasite control was effective, and persistent low or intermittent parasitemia was seen in the chronic phase, at 120 and 150 dpi (Fig 1A). A kinetic study in Colombian-infected C57BL/6 mice showed that most of the behavioral changes are established at 120 dpi [18]. Based on these findings, we settled experiments to test the effects of Bz and PTX initiating therapies at 120 dpi (Fig 1B), allowing us to evaluate their effects in preventing the onset of or reversing behavioral and cognitive changes. At 150 dpi, all C57BL/6 mice infected with 100 bt forms of the Colombian strain survived. As expected, Bz therapy reduced parasitemia and parasite load in the cerebral cortex and hippocampus (Fig 1C, D). PTX treatment did not affect parasitemia (Fig 1C) but reduced parasitism in the cerebral cortex and hippocampus (Fig 1D). Moreover, the Bz + PTX combination controlled parasitemia and parasite burden in the CNS areas (Fig 1C, D).

### Effects of therapies with PTX, Bz, and Bz + PTX on behavioral and cognitive changes in chronically *T. cruzi*-infected mice

**Fig 2:**
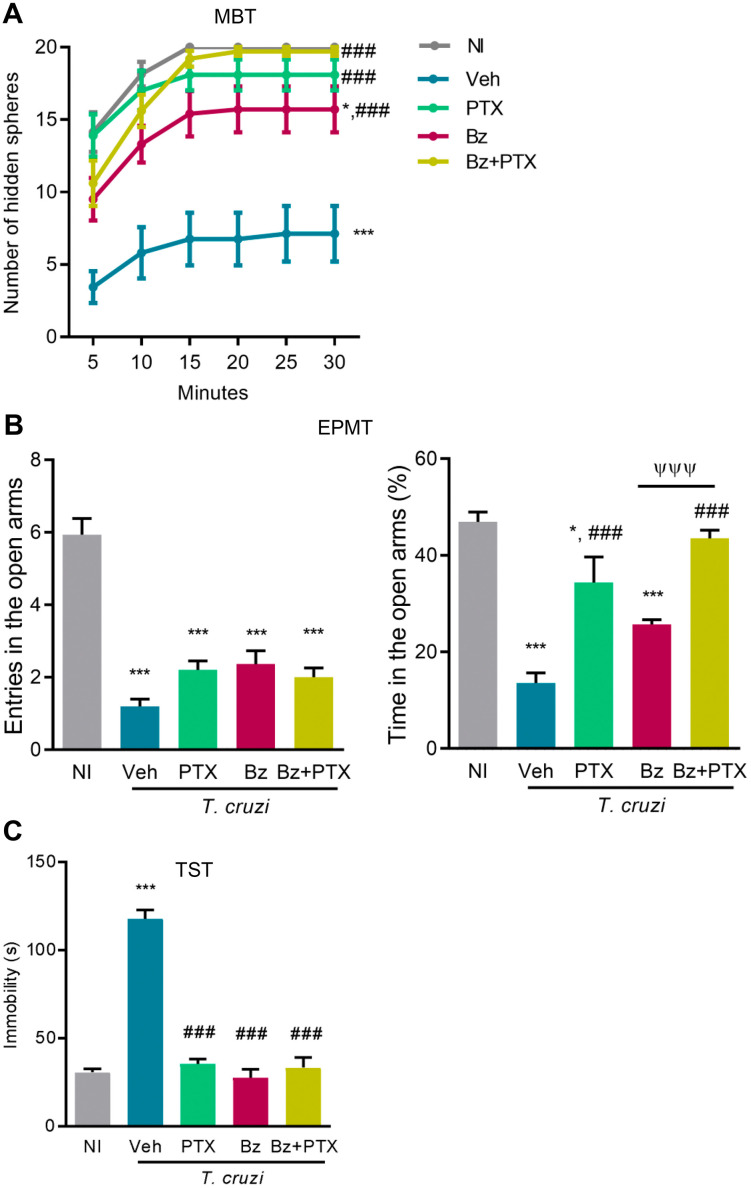
Behavioral changes in chronically *Trypanosoma cruzi*-infected mice are improved by PTX, Bz, and Bz + PTX therapies. C57BL/6 mice were infected with 100 bt of the Colombian *T. cruzi* strain. Mice received PTX, Bz, or Bz + PTX therapies daily from 120-150 dpi. At 150 dpi, mice were analyzed using standardized behavioral tests. (A) Number of hidden spheres in a 30-minute marble burying test (MBT). (B) Number of entries and time spent in the open arms in an elevated plus maze test (EPMT). (C) Time of immobility in the tail-suspension test (TST). Color code: Grey bars indicate non-infected controls (NI), blue bars show Veh-treated, green bars indicate PTX-treated, pink bars show Bz-treated, and yellow bars indicate Bz + PTX-treated infected mice. The data are shown as the means ± SD. *, *p* < 0.05 and ***, *p* < 0.001, *T. cruzi*-infected compared with NI controls. ^###^, *p* < 0.001, treatments compared with Veh-treated *T. cruzi*-infected mice. ^ψψψ^, *p* < 0.001, Bz + PTX-treated compared with Bz-treated *T. cruzi*-infected mice.

At 150 dpi, in contrast with the innate compulsive behavior (assessed by MBT) detected in NI mice, all Veh-treated *T. cruzi*-infected mice showed reduced ability to bury aversive materials (marbles, in this case) present in their environment (*p* < 0.001; Fig 2A). Compared with Veh-treated *T. cruzi*-infected mice, the three treatment schemes ameliorated the innate compulsive behavior (*p* < 0.001; Fig 2A). However, only Bz + PTX therapy completely abrogated this alteration, as noticed after 20 minutes of the test (*p* < 0.001; Fig 2A). Compared with NI controls, Veh-treated infected mice showed a reduced number of entries and time spent in the open arms in the EPMT (*p* < 0.001), suggestive of anxiety-like behavior. After the three therapeutic schemes, the number of entries in the open arms remained reduced (Fig 2B). The time spent in the open arms of the labyrinth remained reduced in Bz-treated mice, while it was partially restored by PTX therapy (*p* < 0.001). Moreover, Bz + PTX therapy completely restored the time spent in the open arms, compared to Veh- and Bz-treated infected mice (*p* < 0.001; Fig 2B). At 150 dpi, Veh-treated infected mice showed increased immobility time in TST (*p *< 0.001). Significantly, the three therapeutic schemes reduced the time of immobility (*p *< 0.001), now resembling NI controls (Fig 2C), supporting that in chronically infected mice, depressive-like behavior was inhibited by Bz and PTX administered as mono and combined therapies.

**Fig 3:**
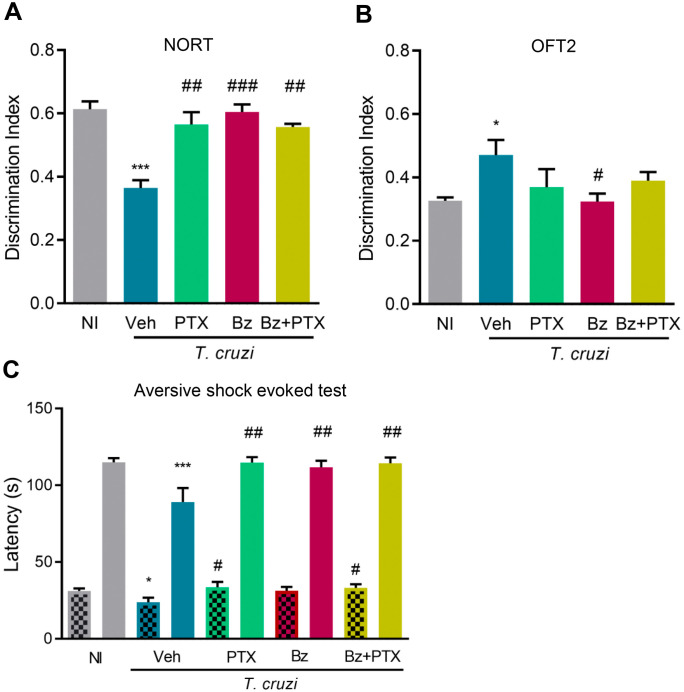
Cognitive alterations in chronically *Trypanosoma cruzi*-infected mice are resolved by PTX, Bz, and Bz + PTX therapies. C57BL/6 mice were infected with 100 bt of the Colombian *T. cruzi* strain. Mice received Veh, PTX, Bz, or Bz + PTX therapies daily from 120-150 dpi. At 150 dpi, mice were analyzed using standardized cognitive tests. (A) Discrimination index in the novel object recognition memory task (NORT); Discrimination index = time exploring the novel object/ (time exploring the novel object + time exploring the familiar object). (B) Discrimination index in the two-day exposition to the open field test, registering the crossed lines (OFT2); Discrimination index = number of crossed lines day 2/ (number of crossed lines day 1 + number of crossed lines day 2). (C) Latency (s) in the training (ASET1, striped bars) and test (ASET2, plain bars) sessions of the aversive shock-evoked test (ASET). Results representative of two independent experiments. Color code: Grey bars indicate non-infected controls (NI), blue bars show Veh-treated, green bars indicate PTX-treated, pink bars show Bz-treated, and yellow bars indicate Bz + PTX-treated infected mice. The data are shown as the means ± SD. *, *p* < 0.05 and ***, *p* < 0.001, *T. cruzi*-infected compared with NI controls. ^#^, *p* < 0.05, ^##^, *p* < 0.01 and ^###^, *p* < 0.001, treatments compared with Veh-treated *T. cruzi*-infected mice.

Memory deficits are sequentially established in chronically *T. cruzi*-infected mice (novel object recognition, spatial habituation, and aversive shock evoked memory) [17,18]. At 150 dpi, NORT revealed a deficit of object recognition in Veh-treated infected mice (*p *< 0.001), a condition inhibited by the three therapeutic schemes (Fig 3A). At 150 dpi, Veh-treated infected C57BL/6 mice presented disturbed spatial habituation memory at the OFT2 (*p *< 0.05), compared with age-matched NI controls, a trait completely abrogated by Bz therapy (*p *< 0.05), while partially ameliorated by PTX and Bz + PTX therapies (Fig 3B). Lastly, compared with age-matched NI controls, Veh-treated infected mice showed decreased latency in the training session for learning and memory consolidation at the ASET1 (*p* < 0.05) and test session for memory recall at ASET2 (*p* < 0.001) as shown in Fig 3C, findings compatible with memory dysfunction. PTX and Bz + PTX therapies improved the latency in the training session (*p *< 0.05). Interestingly, the three therapeutic schemes ultimately hampered (*p *< 0.01) memory impairment in the test session (Fig 3C). Altogether, these therapeutic schemes improved or resolved the behavioral and mnemonic alterations in chronically *T. cruzi*-infected mice.

### PTX and Bz monotherapies and combined Bz + PTX therapy ameliorate neurochemical changes in chronically *T. cruzi*-infected mice

**Fig 4:**
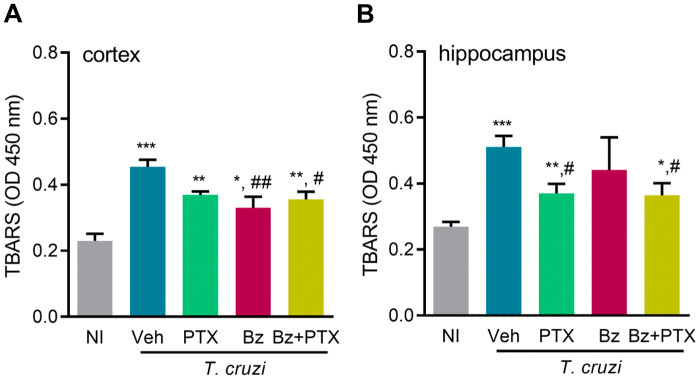
PTX, Bz, and Bz + PTX therapies ameliorate oxidative stress in chronically *Trypanosoma cruzi*-infected mice. C57BL/6 mice were infected with 100 bt of the Colombian strain. Mice received PTX, Bz, or Bz + PTX therapies daily from 120-150 dpi. Oxidative stress, revealed by the detection of TBARS, was detected in the (A) cerebral cortex and (B) hippocampus of the brain tissue. Representative data of two independent experiments. Color code: Grey bars indicate non-infected controls (NI), blue bars show Veh-treated, green bars indicate PTX-treated, pink bars show Bz-treated, and yellow bars indicate Bz + PTX-treated infected mice. The data are shown as the means ± SD. *, *p* < 0.05, **, *p* < 0.01 and ***, *p* < 0.001, *T. cruzi*-infected compared with NI controls. ^#^, *p* < 0.05 and ^##^, *p* < 0.01, treatments compared with Veh-treated *T. cruzi*-infected mice.

At 150 dpi, increased TBARS levels, revealing lipid peroxidation and ultimately oxidative stress, were detected in the cerebral cortex (*p *< 0.001) and hippocampus (*p *< 0.001) of Veh-treated infected mice, compared with NI controls (Fig 4A, B). Administration of Bz (*p *< 0.01) and Bz + PTX (*p *< 0.05) partially reduced the TBARS levels in the cerebral cortex (Fig 4A). In contrast, PTX (*p *< 0.05) and Bz + PTX (*p *< 0.05) therapies were more effective in the hippocampus areas (Fig 4B).

**Fig 5:**
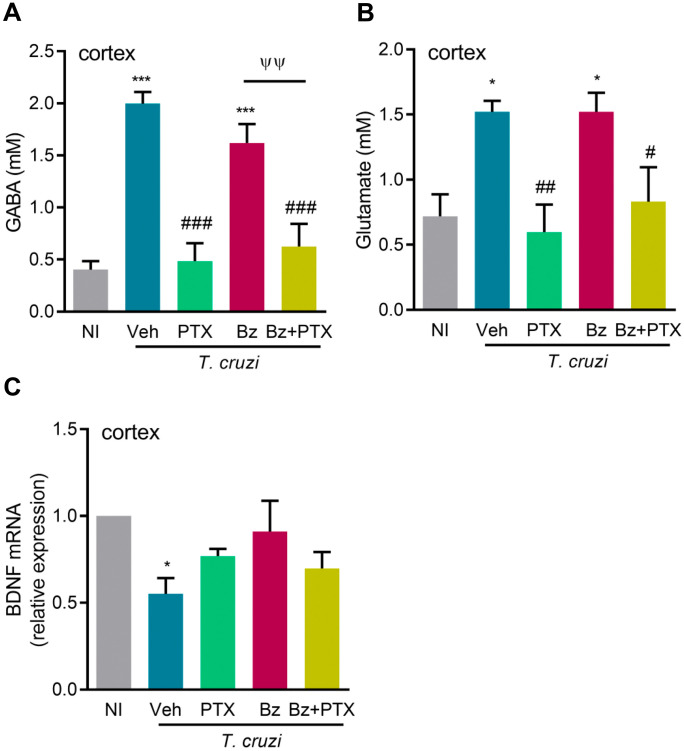
PTX- and Bz-based therapies improve the disbalance of neurotransmitters and BDNF in *Trypanosoma cruzi*-infected mice. C57BL/6 mice were infected with 100 bt of the Colombian *T. cruzi* strain. Mice received PTX, Bz, or Bz + PTX therapies, daily from 120-150 dpi. (A) Using the cerebral cortex, nuclear magnetic resonance analyzed GABA and (B) glutamate. (C) BDNF mRNA expression in the cerebral cortex. Color code: Grey bars indicate non-infected controls (NI), blue bars show Veh-treated, green bars indicate PTX-treated, pink bars show Bz-treated, and yellow bars indicate Bz + PTX-treated infected mice. The data are shown as the means ± SD. *, *p* < 0.05 and ***, *p* < 0.001, *T. cruzi*-infected compared with NI controls. ^#^, *p* < 0.05, ^##^, *p* < 0.01 and ^###^, *p* < 0.001, treatments compared with Veh-treated *T. cruzi*-infected mice. ^^ψψ^^, *p* < 0.01, Bz + PTX-treated compared with Bz-treated *T. cruzi*-infected mice.

Neurochemical changes linked to canonical neurotransmitter and neurotrophin pathways are mainly found in the cerebral cortex of chronically *T. cruzi*-infected mice [18]. Here, we show increased concentrations of GABA (*p *< 0.001) and glutamate (*p *< 0.05) in extracts of the cerebral cortex of chronically Veh-treated *T. cruzi*-infected C57BL/6 mice, compared with NI controls (Fig 5A, B). At 150 dpi, PTX and Bz + PTX therapies, but not Bz treatment, reduced GABA (*p *< 0.001) and glutamate (*p *< 0.01; *p *< 0.05) concentrations in the cerebral cortex of infected mice (Fig 5A, B). Compared with NI controls, a decreased BDNF mRNA expression was observed in the cerebral cortex (*p *< 0.05) of Veh-treated infected mice at 150 dpi (Fig 5C). In mice that received the three therapeutic schemes, BDNF expression was partially recovered, with tissues resembling NI controls (Fig 5C).

### The systemic inflammatory profile is more efficiently regulated by Bz-based therapies in chronically *T. cruzi*-infected mice

**Fig 6:**
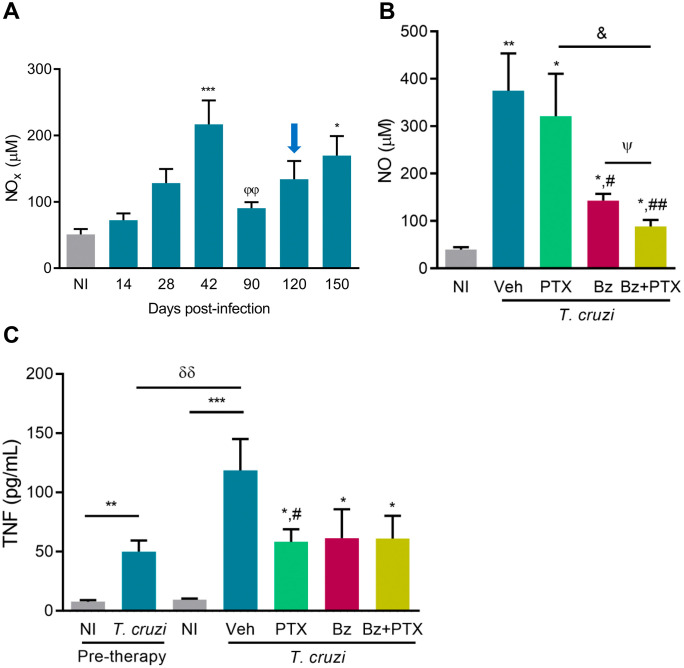
Serum NO and TNF levels are regulated by Bz-based therapies in chronically *Trypanosoma cruzi*-infected mice. C57BL/6 mice were infected with 100 bt of the Colombian *T. cruzi* strain. Sera were collected from 14-150 dpi. Groups of mice received PTX, Bz, or Bz + PTX therapies daily from 120-150 dpi. (A) Kinetic of serum NO concentrations. (B) Serum NO concentrations were measured after therapeutic schemes at 150 dpi. (C) Serum TNF concentrations were determined pre-therapy (120 dpi) and after therapeutic schemes at 150 dpi. Color code: Grey bars indicate non-infected controls (NI), blue bars show not-treated or Veh-treated, green bars indicate PTX-treated, pink bars show Bz-treated, and yellow bars indicate Bz + PTX-treated infected mice. The data are shown as the means ± SD. ^ϕϕ^, *p* < 0.01, 90 dpi compared with 42 dpi. *, *p* < 0.05, **, *p* < 0.01 and ***, *p* < 0.001, *T. cruzi*-infected compared with NI controls. ^#^, *p* < 0.05 and ^##^, *p* < 0.01, treatments compared with Veh-treated *T. cruzi*-infected mice. ^^ψ^^, *p* < 0.05, Bz + PTX-treated compared with Bz-treated *T. cruzi*-infected mice. ^&^, *p* < 0.05, Bz + PTX-treated compared with PTX-treated *T. cruzi*-infected mice. ^δδ^, *p* < 0.01, *T. cruzi*-infected mice pre-therapy (at 120 dpi) compared with Veh-treated *T. cruzi*-infected mice (at 150 dpi).

Next, we questioned the effects of the therapeutic schemes on the systemic inflammatory profile and its regulatory pathways. The kinetic study revealed a peak of serum NO concentrations coinciding with parasitemia (at 42 dpi). After parasitemia control, NO levels were reduced (at 90 dpi), but as the infection progressed, serum NO levels increased again (at 120 and 150 dpi) (Fig 6A), regardless of parasitemia levels, which became negative or intermittent at these last time points (Fig 1A). At 150 dpi, compared with age-matched NI controls, Veh-treated infected mice showed elevated levels of serum NO (Fig 6B). PTX therapy did not impact NO levels, but Bz treatment and, mainly, Bz + PTX therapy reduced serum NO concentrations in chronically *T. cruzi*-infected mice (Fig 6B, S2 Table). In comparison with NI controls, TNF concentrations in serum were increased in infected mice, at 120 dpi (pre-therapy), and even higher as infection progressed (Veh, post-therapy) (Fig 6C). At 150 dpi, TNF levels in mice receiving PTX, Bz, and Bz + PTX therapies were less elevated than in Veh-treated infected mice. They remained like the cytokine levels detected in infected mice pre-therapy (Fig 6C, S2 Table). Thus, Bz-based therapies are pivotal in regulating the systemic NO and TNF levels in chronically *T. cruzi*-infected mice.

**Fig 7:**
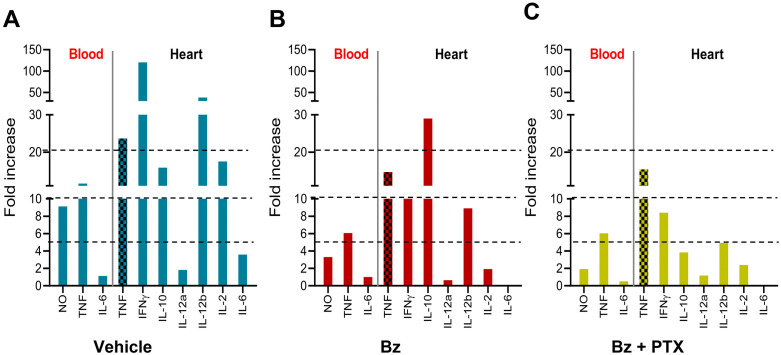
Regulatory role of Bz and Bz + PTX therapies on systemic inflammatory profile in chronically infected mice. C57BL/6 mice were infected with 100 bt of the Colombian *T. cruzi* strain. Mice received PTX, Bz, or Bz + PTX therapies daily from 120−150 dpi. At 150 dpi, blood was collected, NO concentrations were measured, and cytokines were evaluated in serum by CBA. To obtain data on the expression of cytokine genes in the heart tissue of Bz- and Bz + PTX-treated mice, previously published data [37,43] were reanalyzed, focusing on cytokines associated with inflammation (IL-6, IL-10, MCP-1/CCL2, IFN-γ, TNF, and IL-12p70). All data were normalized using serum or tissue samples of matched NI controls of each set of experiments to allow comparative analysis, and data are expressed as a fold increase. (A) Veh-treated, (B) Bz-treated, and (C) Bz + PTX-treated *T. cruzi*-infected mice. Dotted lines were used to facilitate data visualization.

Previous work showed the absence of detectable cytokine gene expression in the CNS but elevated concentrations of cytokines in the serum of chronically *T. cruzi*-infected C57BL/6 mice [18]. Here, we showed increased NO and TNF serum levels 120 dpi. To further explore the idea that the Bz-based therapeutic strategies beneficially act on systemic inflammation, we compared the inflammatory profiles in serum obtained in our present study with findings on cytokine gene expression in the heart tissue of Veh-, Bz- and Bz + PTX-treated mice as a complementary readout of systemic inflammation. For that, we reanalyzed our previous data [37,43], focusing on cytokines associated with inflammation (IL-6, IL-10, MCP-1/CCL2, IFN-γ, TNF, and IL-12p70), normalizing the data using serum or heart tissue samples of matched NI controls. Compared with Veh-treated infected mice (Fig 7A), serum of Bz- and, mainly, Bz + PTX-treated mice showed reduced levels of NO, TNF, and IL-6 (Fig 7B, C). Heart tissue of Veh-treated infected mice showed an elevated fold increase of all analyzed cytokines (Fig 7A). The tissue of Bz-treated infected mice showed reduced expression of all inflammatory cytokine genes, while the regulatory IL-10 cytokine was upregulated (Fig 7B). Crucially, Bz + PTX therapy reduced the expression of all analyzed cytokine genes (Fig 7C). Thus, multiparameter analysis supports that the combined Bz + PTX therapy more efficiently dulls the systemic inflammatory profile detected in chronically *T. cruzi*-infected mice.

Our investigation into the systemic inflammatory profiles of chronically infected mice has been furthered by evaluating the modulatory effects of Bz-based therapy on cytokine expression (Fig 7). The in-depth transcriptomic standing of the interaction between chronic *T. cruzi* infection and the systemic regulation of miRNAs, particularly those implicated in inflammation and neurodegenerative processes, expands upon the influence of Bz-based therapeutic regimens. While we acknowledge that accessing the miRNA transcriptome in brain tissues and serum samples of mice poses a challenge, it emphasizes the significance of analyzing the heart tissue for a comprehensive miRNA profile as a marker of systemic processes. Hence, we have reexamined immune response-related mRNA and miRNA transcriptome data originally detailed by Farani et al. [43,44]. Differentially expressed mRNA (DEGs) and miRNAs (DEMs) were identified through comparative analysis between the Veh-treated group and NI controls, offering insights into potential biomarkers for further studies. Regarding the immune response-related genes identified, we explored the activated canonical pathways, where the Veh-treated group includes ‘Th1 and Th2 Activation Pathway’ and ‘Th1 Pathway’, suggesting an immune response skewed towards T helper cell activation and other pathways such as ‘Neuromodulation Signaling Pathway’ is also significant, pointing to ongoing inflammation and potential neurological modulation in mice infected with *T. cruzi* (S4A Fig). The Bz-treated group exhibited activation of ‘Th1 and Th2 Activation Pathway’, albeit less significantly compared to the Veh-treated group (S4B Fig) and finally, Bz + PTX-treated group revealed remarkable inhibition of pathways related to ‘Th2 Activation’ and ‘Th1 Pathway’, denoting a potential therapeutic effect of the combined treatment in modulating these immune responses, and activation of ‘IL-12 Signaling and Production in Macrophages’ pathway, suggesting a specific immune regulatory impact due to the combined therapy (S4C Fig).

**Fig 8:**
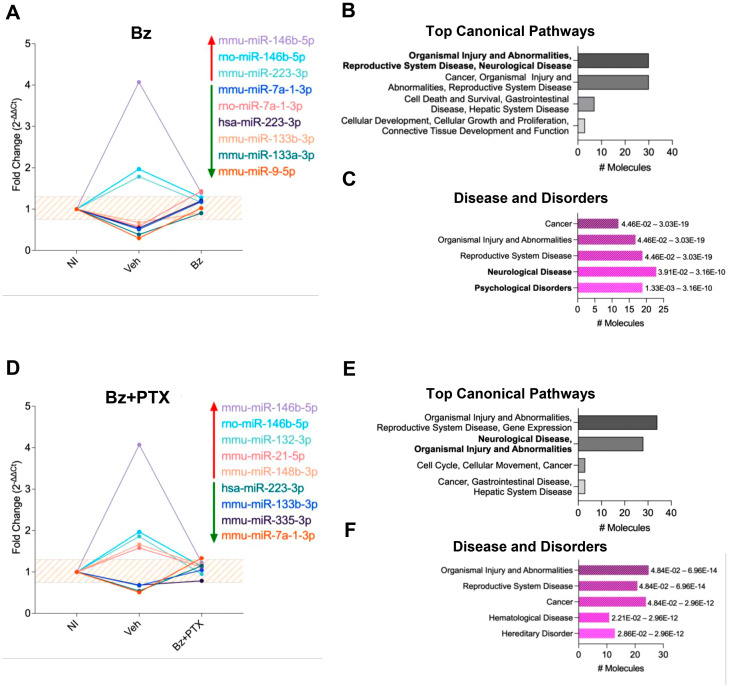
Characterization of miRNA expression profiles and their potential targets upon Bz and Bz + PTX therapy in chronically infected mice. Relative expression was expressed as fold change (2^-ΔΔCt^). miRNAs with a fold change greater than 1.3 in the vehicle group and at the 0.75–1.3 range (orange striped area) in the Bz and Bz + PTX therapy groups were considered to have a restored expression. They were selected for further analysis on IPA software. (A) The expression of miRNAs was altered in the infected group and an expression was restored after Bz therapy. (B) Top canonical activated pathways in the infected group with a restored expression upon Bz therapy. (C) Diseases and disorders pathway analysis. (D) The expression of miRNAs was altered in the infected group and restored upon Bz + PTX therapy. (E) Top canonical activated pathways in the infected group with a restored expression upon Bz therapy. (F) Diseases and disorders pathway analysis. Significant intervals of enrichment are shown on the right of the bars.

We identified 24 DEMs associated with inflammatory and neurodegenerative pathways that were altered in Veh-treated infected mice (S3 Table), 9 of those were restored after Bz therapy – 3 up- and 6 downregulated in *T. cruzi*-infected mice (Fig 8A, S4 Table), and other 9 were restored after Bz + PTX therapy, – 5 up- and 4 downregulated in *T. cruzi-*infected mice (Fig 8D, S5 Table). DEMs ranging from −0.7 to +1.3-fold change underwent functional pathway analysis using Ingenuity Pathway Analysis Software. The elucidation of putative targets for each regulated miRNA through target filter analysis and the core analysis brought to light relevant regulatory pathways. The Top Canonical Pathways impacted by Bz therapy include “organismal injury and abnormalities,” “reproductive system disease” and “neurological disease” (Fig 8B). In contrast, the Disease and Disorders showed alteration of pathways related to “neurological disease” and “psychological disorders” featured prominently in terms of miRNA involvement (Fig 8C). After Bz + PTX treatment, the altered Top Canonical Pathways primarily concerned with “organismal injury and abnormalities,” “gene expression,” and “neurological disease” (Fig 8E). Additionally, Bz + PTX treatment affected regulatory pathways related to Disease and Disorders, wherein “organismal injury and abnormalities,” “reproductive system disease,” and “cancer” were enriched (Fig 8F).

In Veh-treated mice, we selected key biological pathways proposed to underpin CD’s pathogenesis and clinical outcome, including the ‘Inflammation of the central nervous system’, ‘Neuroinflammation signaling pathways’, and ‘Psychological disorders’. A group of DEMs (miR-223-3p, miR-21-5p, miR-182-5p, miR-155-5p, miR-148a-3p, miR-146a-5p, miR-133a-5p, miR-132-3p, let-7d-3p) was found to be linked to the aforementioned pathways (S5 Fig), and identified to directly regulate crucial genes such as NOS2/iNOS (Nitric oxide synthase 2/Inducible nitric oxide synthase) and TLR4 (Toll-like receptor 4). In contrast, Bz-treated groups showed miR-146a-5p and miR-223-3p as key miRNAs in restoring the regulatory networks on ‘Inflammation of central nervous system’ and ‘Psychological disorders’ pathways (S6 Fig). miR-146a-5 p’s regulation of TLR4, CCL5 (Chemokine ligand 5), and SERPINA3 (Serpin peptidase inhibitor, Clade A, Member 3), while miR-223-3p targets PLG (Plasminogen), IL6 (Interleukin-6) highlighting their significance in these processes. The combined Bz + PTX therapy not only reinforced the role of miR-146a-5p targeting IL1R1 (Interleukin-1 receptor 1), IL10 (Interleukin 10), CRP (C-reactive protein) and CXCL8 (CXC-chemokine 8 ligand), but also brought to light the involvement of miR-21-5p, miR-132-3p, and miR-148a-3p in modulating genes related to ‘Neurodegenerative diseases’ and ‘Central nervous system inflammation’ (S7 Fig). As an example, miR-21-5p was observed to regulate pro-inflammatory cytokines such as IL12A (Interleukin 12A) and TNF (Tumor necrosis factor). Further, miR-132-3p directly regulates the genes GRM3 (Glutamate metabotropic receptor 3) and MMP9 (Matrix metalloproteinase 9), while miR-148a-3p directly regulates the channel KCNJ6 (Potassium inwardly rectifying channel subfamily J member 6) and PD1A3 (protein disulfide-isomerase A3).

### Combined Bz + PTX therapy ameliorates behavioral and cognitive changes regulating the neurochemical changes and systemic inflammatory profile

**Fig 9:**
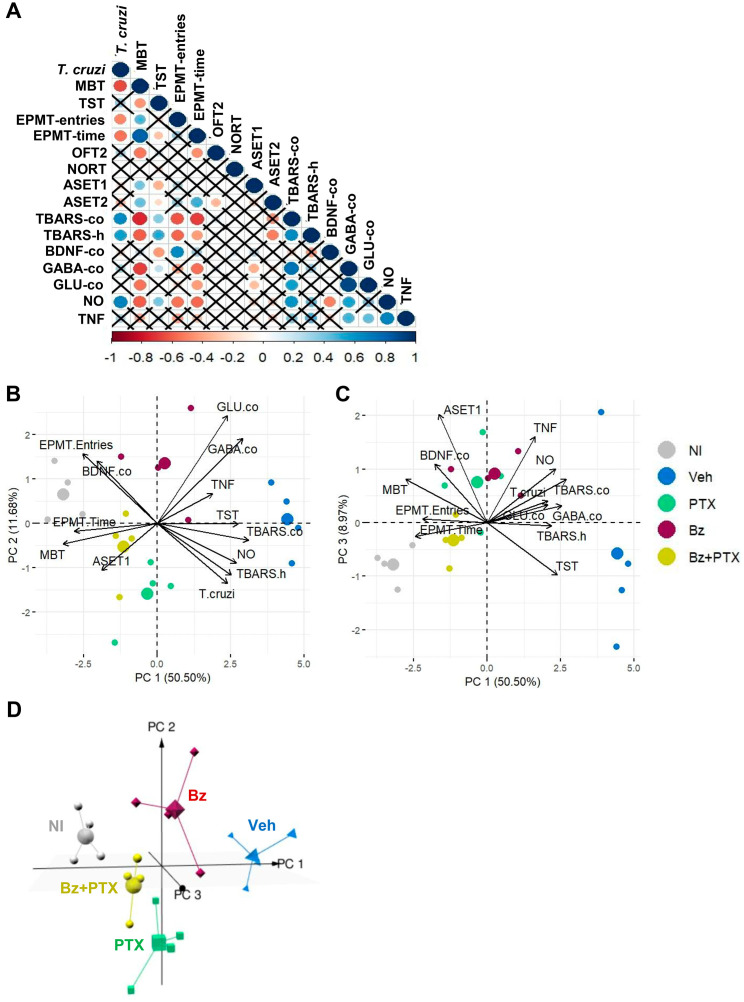
Relationship between parasitemia, neurochemical and immunological stressors and behavioral and cognitive changes in chronically infected mice. (A) A circle represents a significant correlation, and the intensity of the color indicates the strength of this correlation, which is represented in blue (direct correlation, 0 to +1) or red (inverse correlation, 0 to −1). The “X” indicates the absence of a significant correlation between the variables. (B-C) The three first principal components explain 71.15% of the data variance, and the 2D projection of (B) PC1-PC2 and (C) PC1 and PC3 indicate the association between parasite load (*T. cruzi*), serum TNF and NO levels, and neurochemical changes in the CNS, and the behavioral and cognitive changes. (D) The 3D projection, with the three first principal components, shows that NI control group and Bz + PTX-treated *T. cruzi*-infected group show similar behavior and demonstrate the differences between the Veh-treated infected group and NI cluster, and Bz + PTX-treated and Veh-treated cluster.

Next, we ran a multivariate analysis to identify an association between processes and visualize the profile of the groups that received therapies. Initially, we performed a correlation analysis using the Spearman correlation test for data of parasitemia (*T. cruzi*), behavioral and cognitive changes, neurochemical alterations (TBARS, GABA and glutamate, BDNF), and serum inflammatory profiles (TNF, NO), comparing NI controls, Veh-treated, PTX-, Bz- and Bz + PTX-treated *T. cruzi*-infected mice. The correlation analysis (Figs 9A and S8) demonstrated a direct association between *T. cruzi* infection and oxidative stress in the cerebral cortex (TBARS-co) and hippocampus (TBARS-h) and serum NO levels (NO). Further, serum TNF levels were correlated with serum NO levels, the neurotransmitters GABA, and glutamate (GLU) concentrations in the cerebral cortex. Crucially, serum NO levels were directly correlated with oxidative stress (TBARS-co) and GABA concentrations in the cerebral cortex while inversely correlated with BDNF expression in the brain cortex. Regarding behavioral changes, the loss of innate compulsive behavior (MBT – decreased number of marble burying), depressive-like behavior (TST – increased immobility time), and anxiety (EPMT-entries and EPMT-time – reduced number of entries and time in open arms) were associated with the serum NO levels and oxidative stress (TBARS-co and TBARS-h). Likewise, these changes, except for TST, showed a negative correlation with parasitemia (*T. cruzi*); that is, at higher levels of parasitemia, there is a reduction in the evaluated behaviors (decrease in the number of marble burials and a reduction in the number of entries and time in open arms, indicative of loss of innate compulsive behavior and anxiety as parasitemia increases. Further, learning and memory consolidation at the ASET (ASET1) was inversely related to GABA and glutamate concentrations in the cerebral cortex (GABA-co; GLU-co), while memory recall (ASET2) was inversely related to the oxidative stress in the hippocampus (TBARS-h). Interestingly, except for the readout of the memory of object recognition (NORT), the readouts of other behavioral tests were directly or indirectly related, supporting an association between loss of innate compulsive behavior, anxiety, depressive-like behavior, and habituation and aversive memory deficits (Fig 9A). The correlation values and significance levels are provided in S6 and S7 Tables, respectively.

Lastly, to visualize the similarities between groups and identify clusters of differentiation, we performed a PCA using data of behavioral and cognitive changes and immunological and neurochemical abnormalities, comparing NI controls, Veh-treated, and groups treated with the three therapeutic schemes (PTX, Bz, and Bz + PTX). The three-first principal components were used to explain 71.15% of the data variance. As shown in Fig 9B, the 2D projections of the samples using the two-first components, which explain 62.18% of the variance, confirmed the association between infection (*T. cruzi*) levels, increased serum NO levels, and increased TBARS levels in the cerebral cortex and hippocampus. The increase in these variables is correlated with decreased BDNF expression in the cortex and reduced entries into the open arms of the EPMT (anxiety). At the same time, the presence of depressive-like behavior was shown to be related to an increase in the TBARS levels in the cerebral cortex. Also, the increased concentrations of the neurotransmitters GABA and glutamate in the brain cortex were correlated with the increased serum TNF concentrations, which correlated with depressive-like behavior. Moreover, the increase in GABA and glutamate in the brain tissue and TNF in serum are inversely correlated with the decrease in the number of hidden marbles in the MBT, the reduction in the time mice spent in the open arms of the EPMT and the decrease latency in aversive shock evoked test (ASET1) (Fig 9B), sustaining the association of serum TNF concentrations and neurotransmitter changes in the brain tissue with loss of innate compulsive behavior, anxiety and consolidation aversive memory. Additionally, increased parasitemia showed a close relationship with increased TNF and NO serum levels, an increase in TBARS and neurotransmitter levels in the brain cortex, and depressive-like behavior in the group of Veh-treated infected mice (Fig 9C). PC1 and PC3 projection showed similar profiles as in PC1 and PC2 (Fig 9B, C). Moreover, 3D projections, with the three first principal components explaining 71.15% of the variance, confirmed that well-defined clusters were observed separating the Veh-treated infected group, showing completely different profiles than the other groups. At the same time, the NI control group, Bz-treated and Bz + PTX-treated groups were visualized closely, regarding parameters such as decrease in anxiety (EPMT), improvement in innate compulsive behavior (MBT), increase in the consolidation of the aversive memory (ASET1), and increase in the BNDF expression in the cerebral cortex. Indeed, our PCA analysis supports a closer proximity between the Bz + PTX-treated infected group and NI control group (Fig 9D).

## Discussion

Here, we showed the benefits of combining etiological and immunoregulatory therapies to treat a preclinical model of chronic CD. Multivariate analysis revealed that although monotherapeutic schemes with Bz and PTX were efficient, Bz + PTX therapy added advantages in neurochemical and immunological abnormalities, thus explaining its differential impact hampering progression or reversing behavioral and cognitive disorders in chronic *T. cruzi* infection. Lastly, our data opens the way to identify systemic biomarkers linked to mental disorders in CD.

CD patients may suffer from behavioral and cognitive alterations [4–9]. Socio-economical and psychological stressors, conditions most CD patients face [12], may contribute to behavioral and cognitive changes [11]. However, preclinical findings support that biological stressors underpin behavioral (anxiety, depression) and cognitive (learning and memory recall) changes in *T. cruzi* infection [13–17]. Recently, we described that in Colombian *T. cruzi* strain-infected C57BL/6 the onset of behavioral and cognitive changes is sequential, and at 120 dpi, when parasitemia is controlled, most behavioral changes are detected, and mnemonic alterations are partially settled [17,18], opening a window of opportunities to apply therapeutic tools to reverse and/or hamper the progression of these alterations. Here, we used this model of sequential onset of behavioral and cognitive disorders to investigate the participation of the *T. cruzi* parasite and systemic inflammatory profile in these alterations. We used therapeutic schemes based on a currently in use etiological medication (Bz) and an immunoregulatory medication (PTX) repositioning. PTX did not affect parasitemia and when combined therapy, did not disturb the trypanossomicidal effect of Bz, corroborating previous studies focused on heart parasitism [36,37,43,44]. More importantly, the three therapeutic schemes reduced parasite load in the cerebral cortex and hippocampus. Intracellular forms of the parasite persist in different areas of the CNS of chronically infected rats [56] and mice [17,57,58], reproducing findings described in CD patients [59,60]. Further, the reactivation of *T. cruzi* infection in the CNS of chronically infected patients co-infected with HIV linked to neurological alterations [61] is responsive to Bz therapy, whether timely used [62]. These data support the presence of *T. cruzi* parasite in the brain tissue and the accessibility of the CNS to Bz, as demonstrated in a preclinical study [63]. Therefore, the efficacy of Bz in brain tissue was expected, as previous data show that Bz therapy reduced parasite load in the CNS [17] and systemically in the heart tissue and peripheral blood of chronically Colombian-infected mice [37,64]. Although PTX did not affect parasitemia, parasite load in the CNS was lowered in the PTX and Bz + PTX groups. This surprising effect of PTX in monotherapy may be explained by its efficacy in reducing TNF receptor 1 (TNFR1) expression on glial cells, inhibiting the TNF-induced invasion of astrocyte by *T. cruzi* [65], which may disrupt the parasite cycle in brain cells, lowering parasitism consequently. Further, PTX restored TCR expression, preserved interferon-gamma production, and favored *T. cruzi* antigen-specific immune response in splenocytes [36]. These findings allowed us to use the Bz + PTX combined therapeutic strategy to evaluate the impact on behavioral and cognitive disorders in chronic *T. cruzi* infection. The three therapeutic schemes have beneficial effects hampering or reversing behavioral and cognitive disorders. Moreover, Bz + PTX combined therapy had a broader beneficial effect on these changes, particularly innate compulsive behavior, anxiety, and aversive memory. In chronically *T. cruzi*-infected mice, Bz, PTX, and, mainly, Bz + PTX combined therapy have beneficial effects on heart electrocardiographic abnormalities [37]. Thus, this beneficial role in behavioral and cognitive changes may rely on their effects on improving heart capacity in infected mice, potentially linking behavioral changes to cardiac abnormalities. However, this does not seem to be the case, as in elderly CD patients, cognitive changes have been detected in association with positive serology but independently of electrocardiographic changes [10]. Furthermore, recently, we showed that behavioral and cognitive changes were ameliorated by fluoxetine therapy. At the same time, heart abnormalities persisted [18], supporting the dissociation of heart alterations and CNS commitment in the present CD model. In the CNS of chronically *T. cruzi*-infected mice, in the absence of infiltrating inflammatory cells and detectable expression of inflammatory cytokines, other putative stressors were described, such as increased levels of the neuromediators GABA and glutamate, enhanced oxidative stress, and decreased expression of the neurotrophin BDNF [17,18]. Thus, we asked about the impact of therapeutic schemes on these stressors. Crucially, we bring evidence of a complementary effect of Bz and PTX on stressors achieved in the Bz + PTX-treated group. Bz therapy does not affect the elevated GABA and glutamate levels. In contrast, PTX in mono or combined therapy restored the levels of these neuromediators to those found in the non-infected control group. In a non-infectious model, reducing glutamate levels was linked to benefits on depression [66]. Recently, we showed that reducing the elevated levels of GABA and glutamate by fluoxetine therapy ameliorated behavioral and cognitive changes, supporting the participation of these biological stressors in a complex scenario triggered by *T*. *cruzi* infection [18]. Bz + PTX therapy also partially reduced the elevated levels of oxidative stress, revealed by TBARS, in the CNS, as previously shown for Bz in monotherapy [17] and fluoxetine treatment [18], in association with improving memory deficits and behavioral changes. In a model of the acute *T. cruzi* infection, Bz therapy reduced the levels of oxidative stress in the brain cortex but did not affect levels of reactive oxygen species [67]. Oxidative stress has been associated with neurodegenerative disorders and memory deficits [23]. Oxidative stress and lipid peroxidation may significantly reduce BDNF expression [68]. Here, the three therapeutic schemes partially restored the reduced BDNF expression in the brain cortex of chronically infected mice, paralleling the improvement of behavioral and cognitive changes. Thus, our data reinforce the described roles of BDNF deficit in depression [69] and anxiety [20]. Further, BDNF plays crucial physiological roles in maintaining body weight, brain morphological integrity [20] and taking part in biological processes such as neuronal survival, growth, and plasticity, processes crucial for learning and memory formation [21]. Therefore, combined Bz + PTX therapy may emerge as a therapeutic tool of broader relevance that should be further explored.

Bz administration to acutely infected mice hampered depression in the chronic phase of infection [14]. In chronically infected mice, Bz therapy reversed mnemonic alterations while reducing parasite load [17]. However, Bz therapy only partially ameliorates these behavioral changes, supporting the participation of additional stressors. The presence of depressive behavior in arthritis and hepatitis C virus-seropositive patients undergoing immunotherapy with IFNα unveiled a connection between increased systemic inflammation and behavioral disorders [70,71]. Elevated levels of systemic inflammatory cytokines are also associated with neurodegenerative disorders [22]. In CD patients with cardiopathy [28–32] and preclinical models that reproduce aspects of Chagas’ heart disease [36,37,43], a systemic inflammatory profile enriched in NO, TNF, and other cytokines has been associated with the severity of cardiac abnormalities. Thus, the systemic inflammatory profile and other immunological abnormalities, such as the exacerbated T-cell activation [36], became attractive targets for therapeutic intervention to treat CD adjunctly to etiological treatments. In chronically infected mice, studies of proof-of-concept showed that anti-TNF neutralizing antibodies beneficially reversed immunological and cardiac alterations [35] and ameliorated depressive behavior [14], showing a role for TNF as a hub in these processes. In preclinical models of chronic chagasic cardiopathy, PTX therapy also reduced electrical abnormalities and heart dysfunction in mice [36] and improved myocardial perfusion in hamsters [72], without disturbing parasite control. Moreover, PTX reversed immunological abnormalities, such as the upregulation of TNFR1 expression and serum cytokine levels, and improved T-cell-mediated anti-parasite immunity [36], which may reduce parasite load. Thus, indirectly, PTX may contribute to reducing the stimulus of the immune response. Intriguingly, our kinetic study showed that NO serum levels, such as TNF levels, were not associated with parasitism in blood but increased as the disease progressed, as well as the cognitive and behavioral disorders in this model [18]. PTX did not affect NO levels, which were reduced after Bz and, mostly, Bz + PTX combined therapy. *In vitro* TNF stimulation and *T. cruzi* infection of astrocytes increased NO and glutamate concentrations in supernatants [19], which may create a neurotoxic milieu. Indeed, high NO levels have neurotoxic effects in Alzheimer disease [25]. Physiologically, NO has pleiotropic roles in the CNS regulating vascular, immune, and metabolic functions, and as a neuromediator favors memory formation [24]. Thus, the beneficial effects of Bz-based therapies may rely on lowering NO levels.

The three therapeutic schemes equally impacted TNF serum levels, compared with Veh administration. In chronic *T. cruzi* infection, Bz treatment may play a role as an immunoregulator indirectly, controlling parasite growth and, therefore, decreasing the activation of the immune system or directly reducing TNF and oxidative stress, as shown in Bz-treated LPS-elicited macrophages [73]. However, when we compared these findings to our previous data on cytokines mRNA in heart tissue [37,43], as a readout of the systemic inflammatory profile, Bz + PTX was a more effective immunoregulatory strategy than Bz as monotherapy. Thus, the combined scheme may contribute to controlling the parasite load and, indirectly or directly, to a more balanced immune response. These effects may contribute to reducing behavioral and cognitive changes in chronic *T. cruzi* infection, supporting the idea that parasite persistence and parasite-triggered dysregulated immune response are crucially involved in these processes.

Next, we aimed to shed light on systemic biological and molecular pathways and complex interactions of mRNAs and miRNAs (DEGs/DEMs) associated with immune system dysregulation and putatively involved in the behavioral and biological changes in this model, and explored the impact of Bz and, particularly, Bz + PTX therapy. Importantly, due to our limitations in accessing miRNA transcriptome data within brain tissues and serum samples of chronically *T. cruzi*-infected mice, we reanalyzed miRNA transcriptome profiling in heart tissue [44], as a systemic activation indicator. This reanalysis was crucial in our understanding of the pathogenesis, as it provided a unique perspective on the biological pathways, including the ‘Inflammation of the central nervous system’, ‘Neuroinflammation signaling pathways’, and ‘Psychological disorders’, which are among the main pathological processes involved in behavioral and cognitive alterations and could play a role in these clinical signs in CD. We identified genes directly regulated, such as the NOS2/iNOS (Nitric oxide synthase 2/Inducible nitric oxide synthase). This enzyme is a key player in immune response but the increased iNOS expression is also linked to inflammation and tissue damage, and high serum NO levels in chronically *T. cruzi*-infected mice [37] and non-human primates [74]. Thus, the regulation of iNOS expression may explain the reduced serum levels of NO after Bz and, mainly, Bz + PTX therapies. Indeed, reduced iNOS expression in heart tissue was seen in the heart tissue after Bz, PTX, and, mainly, Bz + PTX therapy [37]. Further, among the altered miRNAs related to key biological pathways underpinning the pathogenesis of central nervous disorders, miR-146a-5p, miR-146b-5p, miR-132-3p, miR-21-5p, miR-148b-3p were upregulated, while miR-223-3p, miR-7a-1-3p, miR-133a-3p, miR-133b-3p, m miR-9-5p were downregulated. Considering their roles in biological hubs, the ‘Psychological disorders’-related miR-21-5p, for instance, was seen to regulate pro-inflammatory cytokines like IL12A and TNF, which have been previously established as central to the pathology of Chagas’ heart disease [35,75]. Significantly, the expression of these cytokines was downregulated by Bz and, mainly, Bz + PTX therapy, while expression of miR-21-5p was restored to levels found in non-infected mice. Also, the miRNA transcriptome profiling done in cardiac tissue revealed that the upregulated miR-146b-5p, more than 4-fold increased after *T. cruzi* infection, was revealed another restored miRNA after Bz and Bz + PTX therapy. Target prediction analysis revealed that miR-146a-5p emerges as a key miRNA in ‘Inflammation of the central nervous system’ and ‘Psychological disorders’ pathways, and directly targeting cytokines and chemokines, molecules involved in neurodegenerative disorders [22]. Among the altered miRNAs, for instance, miR-146a-5p has been linked to CNS inflammation and miR-132 with neuronal biological processes such as migration integration, dendritic outgrowth, and synaptic plasticity [76]. Thus, more than controlling individual expression miRNAs, both Bz-based therapies significantly restore the physiological expression of a group of miRNAs crucial for biological pathways related to behavioral and cognitive changes present in chronically *T. cruzi*-infected mice. These results indicate that Bz and Bz + PTX therapies modulate various immune-related pathways differently. The combined therapy offers a more balanced immune response by activating and inhibiting specific pathways. This results from our in-depth transcriptomic analysis, which underscores the nuanced effects of Bz-based therapeutic interventions on miRNA regulation in this preclinical CD model. It highlights their potential as viable targets for further biomarker discovery and therapeutic development. These findings underscore the therapeutic potential of Bz + PTX in modulating critical miRNAs and biological pathways involved in the inflammatory response within the CNS. Such insights pave the way for novel interventions targeting specific miRNAs, which could be a strategic approach to mitigating the onset and progression of behavioral and cognitive changes in CD. For instance, the target prediction analysis has emphasized the relevance of miR-146a-5p in inflammatory and neurodegenerative processes, supporting the advantageous role of Bz + PTX therapy on key miRNAs and biological hubs. Therefore, this data set opens new avenues for research into the molecular mechanisms underlying CD and the cognitive and behavioral changes it induces. These findings may attract groups working with CD patients to explore this idea.

Lastly, we ran a multivariate analysis to identify associations between biological processes, and visualize the similarities between groups, comparing NI controls, Veh-treated, and other infected groups that received therapies. We used data on behavioral and cognitive changes and immunological and neurochemical abnormalities to establish correlations between the variables. An initial correlation study showed that, except for the memory of object recognition, there is an association between loss of innate compulsive behavior, anxiety, depressive-like behavior, habituation and aversive memory deficits. Such results are in line with our previous study, showing a sequential establishment of behavioral and cognitive changes [18], suggesting that these alterations may have common triggers and/or amplifiers/perpetuators. Thus, a therapeutic intervention that ameliorates putatively shared pathogenic biological processes may simultaneously impact most behavioral and cognitive alterations. These findings are crucial as serum NO levels were directly correlated with oxidative stress and an increase in the neurotransmitter GABA while inversely correlated with BDNF expression in the brain cortex. TNF serum levels were directly correlated with NO levels in serum and GABA/Glu in the cerebral cortex. Further, our data suggest a direct role of the intensity of blood parasitism in behavioral changes such as loss of compulsive innate behavior and anxiety. Our data also show that behavioral changes such as loss of compulsive innate behavior (MBT), anxiety (EPMT), and depression (TST) are correlated with serum NO levels and oxidative stress in the CNS while learning and memory consolidation (ASET1) is inversely related to GABA and glutamate levels. Memory recall (ASET2) is inversely associated with oxidative stress, therefore, supporting beneficial aspects of the proposed therapeutic strategies. To visualize the similarities between experimental groups and identify clusters of differentiation, we performed a PCA analysis using data on behavioral and cognitive changes and immunological and neurochemical abnormalities, comparing all groups simultaneously. Interestingly, the 2D projections of the samples using the two first components reinforce the relation between parasite load and increased serum NO levels and oxidative levels (TBARS) in brain areas, and these variables were correlated with the decrease of BDNF expression and anxiety (EPMT). All at once, depressive-like behavior was related to an increase in the TBARS levels in the cerebral cortex and serum TNF concentrations. Furthermore, increased concentrations of the neurotransmitters (GABA and glutamate) in the brain cortex were correlated with the increased serum TNF concentrations, which in turn was correlated with depressive-like behavior (TST), loss of innate compulsive behavior (MBT), anxiety (EPMT) and memory acquisition and consolidation (ASET1). Moreover, PC1 and PC3 projections showed similar profiles as those of PC1 and PC2. Notably, 3D projections confirmed well-defined clusters, separating the Veh-treated *T. cruzi*-infected group from groups submitted to therapies. Fatefully, this analysis revealed that all three therapeutic schemes have beneficial effects. Moreover, Bz + PTX therapy added advantages in controlling neurochemical and immunological abnormalities, showing a close relation with the non-infected mice group. Thus, Bz + PTX therapy differentially impacts behavioral and cognitive changes in chronically *T. cruzi*-infected mice.

Altogether, these data reinforce that in chronic infection (*T. cruzi*), systemic inflammatory abnormalities are associated with canonical alterations in the CNS such as elevation of oxidative stress and neurotransmitters (as Glu) and reduction of neurotrophins (as BDNF) in the CNS, as previously described in non-infectious neurodegenerative disorders such as Alzheimer disease [19,25] and behavioral changes such as depression [21]. Again, our findings with therapeutic strategies, particularly the Bz + PTX association, reduce immunological and neurochemical abnormalities and gain strength beyond CD. Oxidative stress has been linked with age-related abnormalities of the CNS [77]. Recent studies proposed using PTX to treat neurodegenerative diseases based on its antioxidative properties [40,41]. Also, independently of its anti-parasitic activity, Bz shows nfr2-linked antioxidative [73] and NF-kB-related anti-inflammatory [78] actions. Altogether, our data reinforce that in CD, a disease in which clinical signs are mostly related to aging [79], the chronic systemic inflammatory process, and oxidative stress and neurochemical changes in the CNS may trigger or accelerate aging-related alterations, leading to sequential behavioral and cognitive abnormalities, as we showed in the preclinical model of CD [18]. Our findings uphold that Bz- and PTX-based antioxidant and anti-inflammatory strategies, combined in the Bz + PTX therapy, may revert these disorders, unveiling a new therapeutic strategy to deal with mental disorders in this infectious condition. More broadly, here we propose that the combined Bz + PTX therapeutic strategy may find room to be tested in preclinical models aiming to reposition medicaments to treat non-infectious behavioral and neurodegenerative disorders.

## Limitations

Although animal models are widely used in the study of diseases, the data generated with our preclinical murine model may suffer from the limitation of not being fully transposable to humans. Our experiments used female mice, considering the gonadal influence on parasitic infection, particularly on *T. cruzi* infection [80], and that sex hormones may impact on the response to the proposed therapeutic schemes, these should also be tested in male mice. Although CD is widely studied through this model, physiological and immunological differences may limit the generalization of the findings to the human clinical context. Although limited in the number of samples analyzed per group due to the high number of experimental groups and the costs of performing PCR analyses in two areas of the CNS, our data consistently support that the three therapeutic regimens did not aggravate parasitism and, moreover, reduced the parasite load in the CNS. As mentioned above, our study only used the miRNA transcriptome of cardiac tissue of the same mice, which was evaluated as a proxy for systemic inflammation, which may not fully reflect the inflammatory profile of other affected tissues, especially the CNS. Our studies were limited by the number of mice approved by the ethical committee, the cost of all performed experiments, and our limitations in accessing miRNA transcriptome data within the same brain tissues and serum samples of the chronically *T. cruzi*-infected mice used to access parasite load, a series of neurochemical changes and systemic immune dysregulation. Direct analysis of miRNAs in the CNS, which shows hundreds of tissue-specific miRNAs [81], would be ideal for understanding the role of these miRNAs in neuroinflammatory processes and the modulation of neurochemical and behavioral and cognitive alterations observed in our CD model.

## Supporting information

S1 Fig Workflow of the experimental protocol. The number of C57BL/6 mice non-infected (NI) and infected with 100 bt forms of the Colombian strain (*T. cruzi*) used perform a kinetic study (14–150 days postinfection, dpi) for immunological analysis. Independent experiments were performed to effects of vehicle (Veh) and benznidazole (Bz) and pentoxiphyline (PTX), as mono or combined therapy (120–15 dpi), and assess the behavioral and cognitive profiles, and biological stressors. Two independent experiments were performed.
(TIF)

S2 Fig Reportable range of qPCR assays targeting *T. cruzi* satDNA (A) and mouse GAPDH (B). TaqMan qPCR assays were carried out from a serial dilution (1:10) of DNA extracted from brain tissue spiked with *T. cruzi*, ranging from 10^5^ to 1 parasite equivalents (A) and from 100 to 10^−3^ brain tissue equivalents (mg) (B). The standard curve parameters, such as qPCR efficiency and coefficient of determination (r^2^), are shown at the bottom left of the graphics.
(TIF)

S3 Fig Body weight and relative spleen weight of *T. cruzi*-infected mice. C57BL/6 mice were infected with 100 bt of the Colombian *T. cruzi* strain. A sorted group of mice were analyzed at 120 dpi. Groups of mice received Veh, PTX, Bz, or Bz + PTX therapies daily from 120–151 dpi. At 120 and 152 dpi (“150 dpi”), mice were weighed, euthanized, and spleens were collected and weighed. (A) Body weight (g). (B) Relative spleen weight = spleen weight (mg)/ body weight (g).
(TIF)

S4 Fig Top canonical pathways. Twenty top canonical pathways activated (intensity depicted in shades of orange) or inhibited (intensity depicted in shades of blue) by the altered immune response genes in the (A) Vehicle, (B) Bz and (C) Bz + PTX group.
(TIF)

S5 Fig Target prediction analysis for the vehicle group. (A) Inflammation of central nervous system pathway. (B) Neuroinflammation signaling pathway. (C) Psychological disorders pathway. The red color indicates miRNA upregulation. Complete lines indicate a direct relationship, while dashed lines indicate an indirect relationship.
(TIF)

S6 Fig Target prediction analysis for the Bz-treated group. (A) Inflammation of central nervous system pathway. (B) Psychological disorders pathway. The red color indicates miRNA upregulation. Complete lines indicate a direct relationship, while dashed lines indicate an indirect relationship.
(TIF)

S7 Fig Target prediction analysis for the Bz + PTX-treated group. (A) Inflammation of central nervous system pathway. (B) Psychological disorders pathway. The red color indicates miRNA upregulation. Complete lines indicate a direct relationship, while dashed lines indicate an indirect relationship.
(TIF)

S8 Fig Correlation analysis, Data show only the values of the correlations that were significant (p < 0.05). Spearman correlation significance test was applied.
(TIF)

S1 Table Data used to build graphs and figures. (DOCX)

S2 Table Pre-therapy and pos-therapy serum concentrations of cytokines and NO. (DOCX)

S3 Table List of up- or downregulated microRNAs (1.3-fold change) in the vehicle-treated group. (DOCX)

S4 Table List of up- or downregulated microRNAs restored with Bz treatment. (DOCX)

S5 Table List of up- or downregulated microRNAs restored with Bz + PTX treatment. (DOCX)

S6 Table Values of observed correlations between behavioral, cognitive and neurochemical variables. (DOCX)

S7 Table P-values of observed correlations between behavioral, cognitive and neurochemical variables. (DOCX)

S1 Checklist Experimental Author´s Checklist. (PDF)
