# Supplementary figures and images for: Combined benznidazole and pentoxifylline therapy improves behavioral and cognitive changes in association with the regulation of systemic inflammatory profile in chronic experimental Chagas disease
Source: PLoS One. 2025 Nov 14;20(11):e0334708. doi: 10.1371/journal.pone.0334708 (PMC12617855; doi:10.1371/journal.pone.0334708)

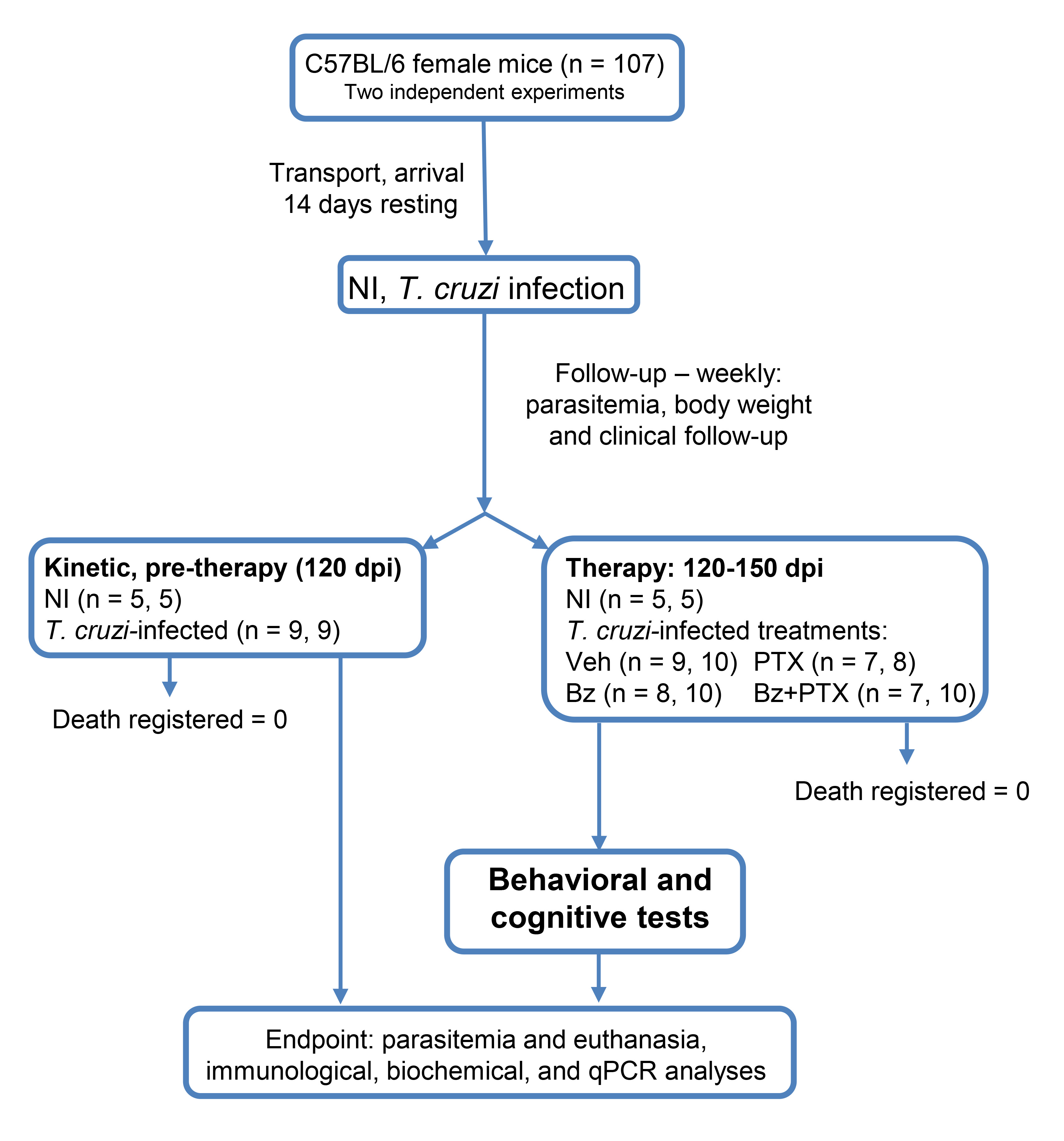

Supplement: S1 Fig — The number of C57BL/6 mice non-infected (NI) and infected with 100 bt forms of the Colombian strain (T. cruzi) used perform a kinetic study (14–150 days postinfection, dpi) for immunological analysis. Independent experiments were performed to effects of vehicle (Veh) and benznidazole (Bz) and pentoxiphyline (PTX), as mono or combined therapy (120–15 dpi), and assess the behavioral and cognitive profiles, and biological stressors. Two independent experiments were performed. (TIF) [file pone.0334708.s001.tif]

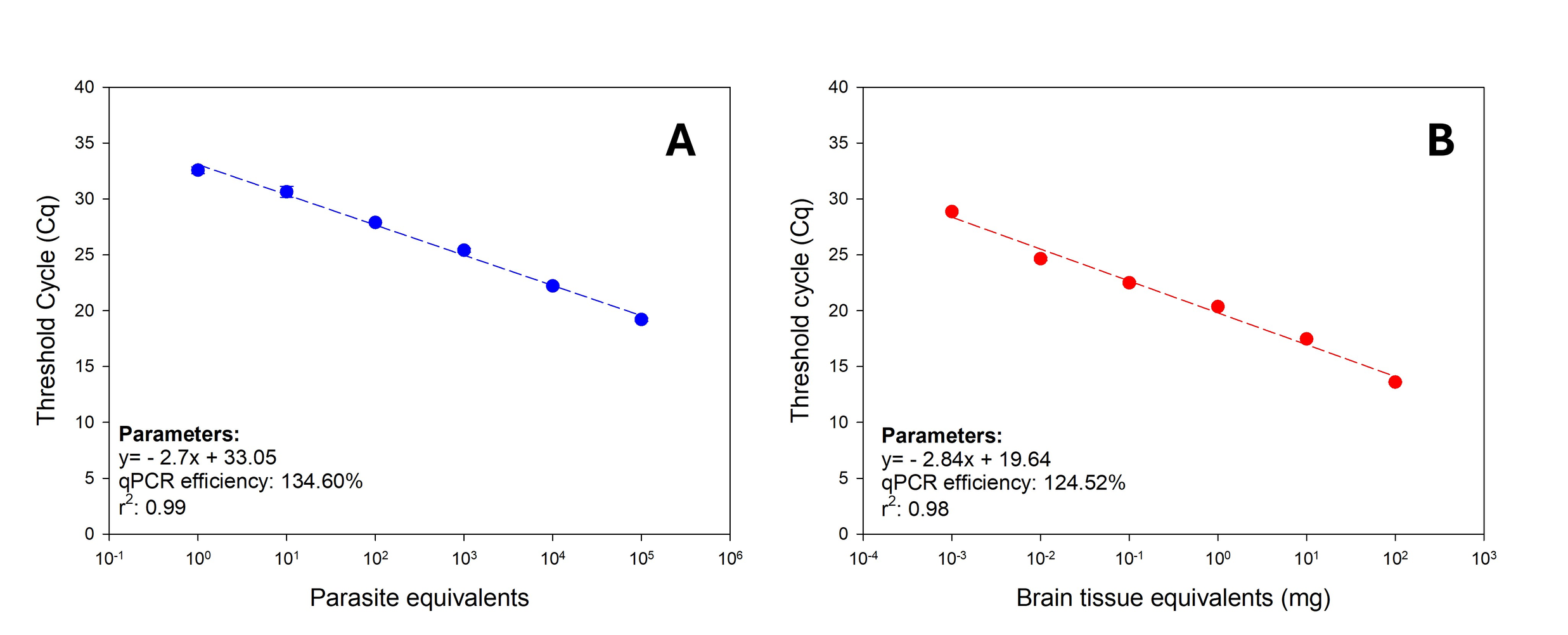

Supplement: S2 Fig — TaqMan qPCR assays were carried out from a serial dilution (1:10) of DNA extracted from brain tissue spiked with T. cruzi, ranging from 105 to 1 parasite equivalents (A) and from 100 to 10−3 brain tissue equivalents (mg) (B). The standard curve parameters, such as qPCR efficiency and coefficient of determination (r2), are shown at the bottom left of the graphics. (TIF) [file pone.0334708.s002.tif]

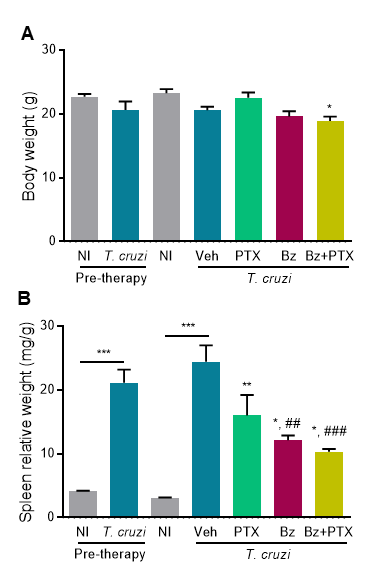

Supplement: S3 Fig — C57BL/6 mice were infected with 100 bt of the Colombian T. cruzi strain. A sorted group of mice were analyzed at 120 dpi. Groups of mice received Veh, PTX, Bz, or Bz + PTX therapies daily from 120–151 dpi. At 120 and 152 dpi (“150 dpi”), mice were weighed, euthanized, and spleens were collected and weighed. (A) Body weight (g). (B) Relative spleen weight = spleen weight (mg)/ body weight (g). (TIF) [file pone.0334708.s003.tif]

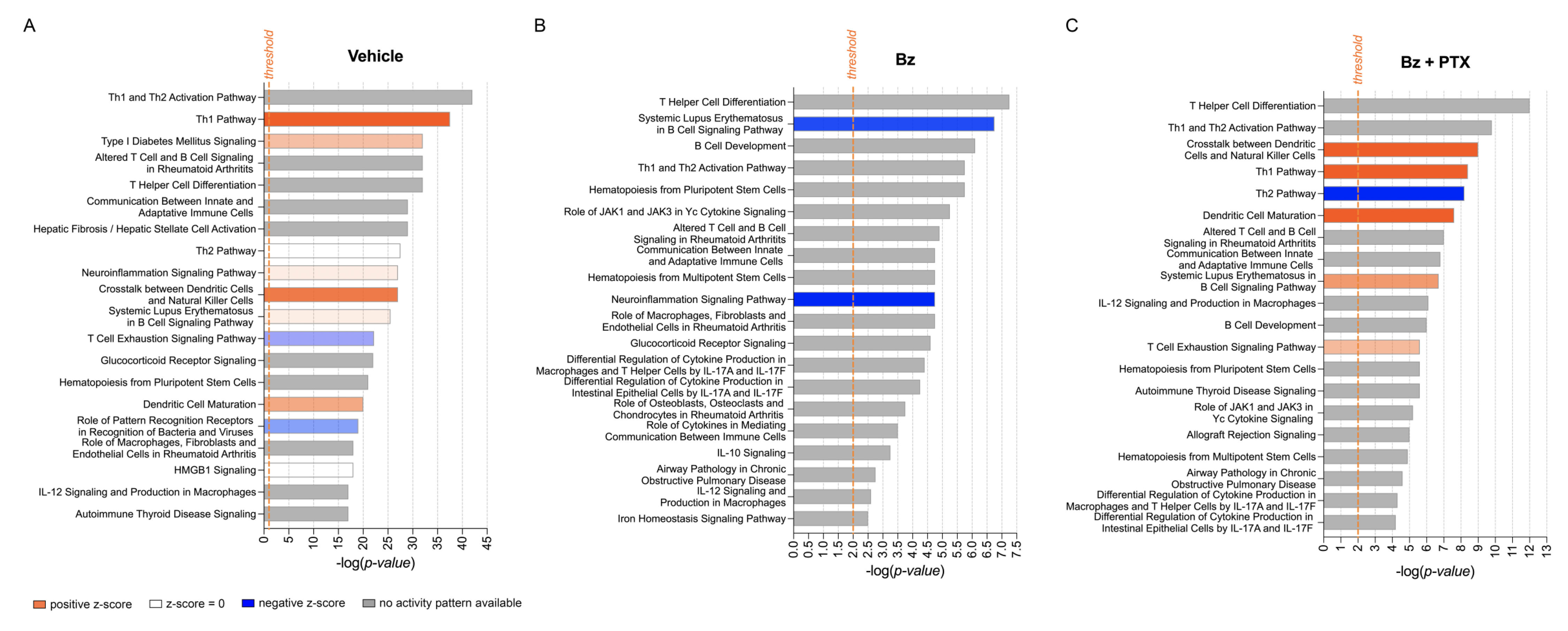

Supplement: S4 Fig — Twenty top canonical pathways activated (intensity depicted in shades of orange) or inhibited (intensity depicted in shades of blue) by the altered immune response genes in the (A) Vehicle, (B) Bz and (C) Bz + PTX group. (TIF) [file pone.0334708.s004.tif]

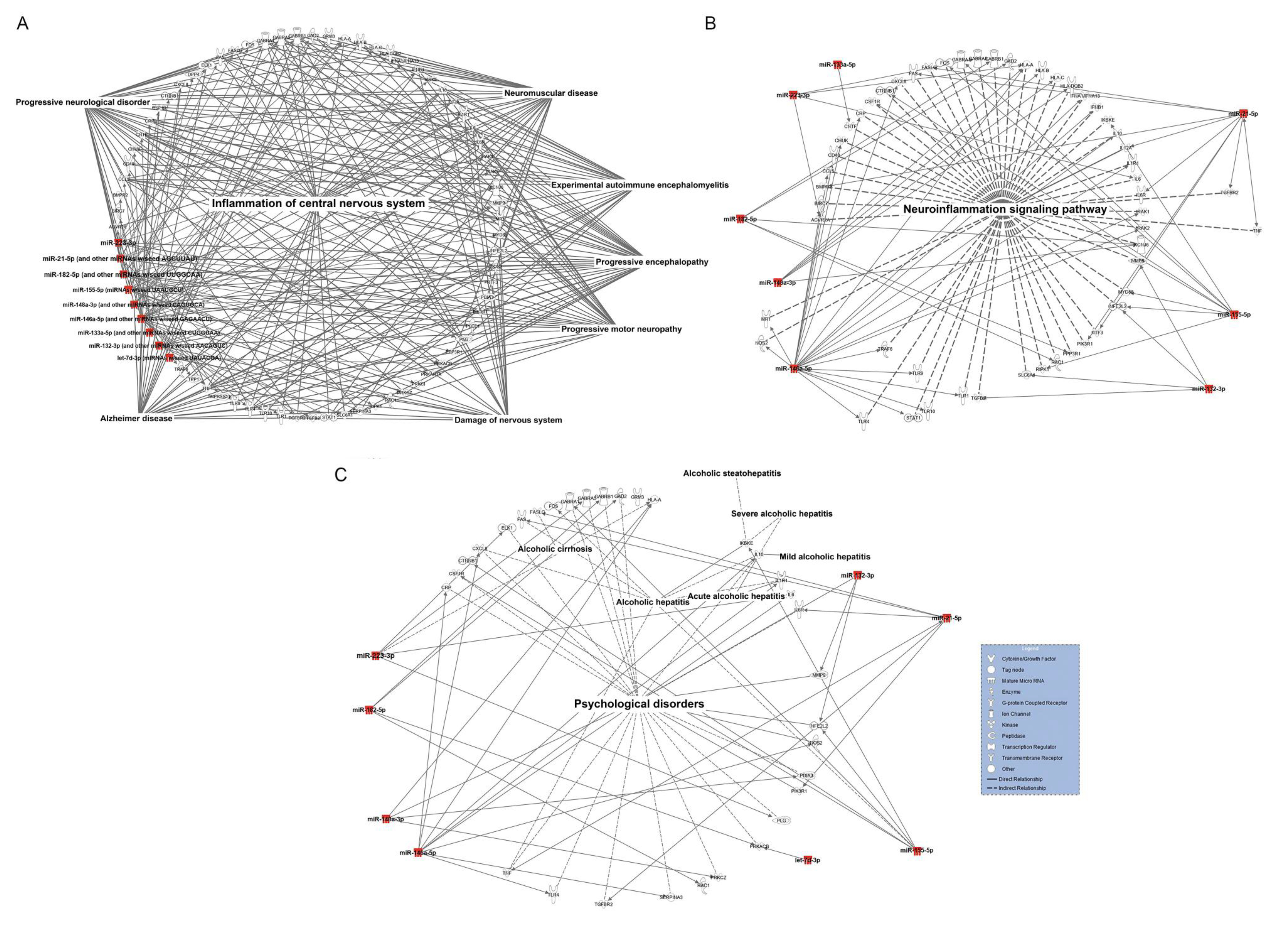

Supplement: S5 Fig — (A) Inflammation of central nervous system pathway. (B) Neuroinflammation signaling pathway. (C) Psychological disorders pathway. The red color indicates miRNA upregulation. Complete lines indicate a direct relationship, while dashed lines indicate an indirect relationship. (TIF) [file pone.0334708.s005.tif]

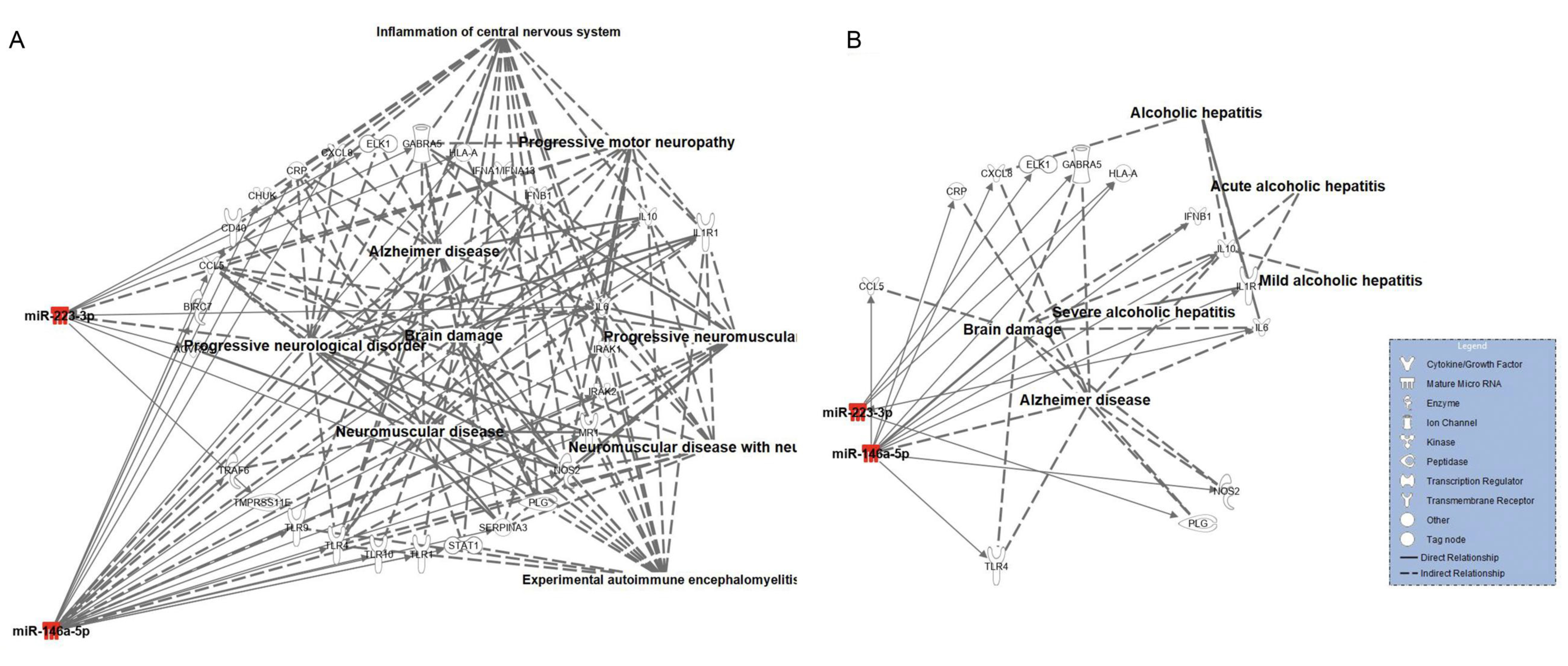

Supplement: S6 Fig — (A) Inflammation of central nervous system pathway. (B) Psychological disorders pathway. The red color indicates miRNA upregulation. Complete lines indicate a direct relationship, while dashed lines indicate an indirect relationship. (TIF) [file pone.0334708.s006.tif]

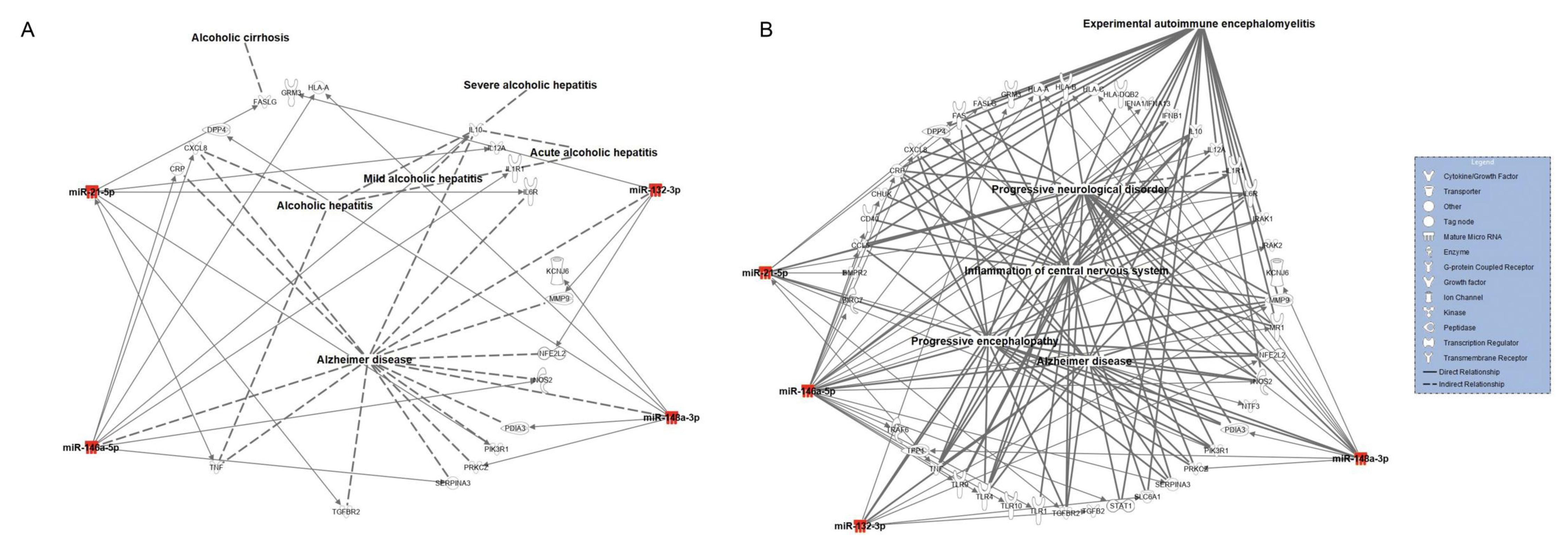

Supplement: S7 Fig — (A) Inflammation of central nervous system pathway. (B) Psychological disorders pathway. The red color indicates miRNA upregulation. Complete lines indicate a direct relationship, while dashed lines indicate an indirect relationship. (TIF) [file pone.0334708.s007.tif]

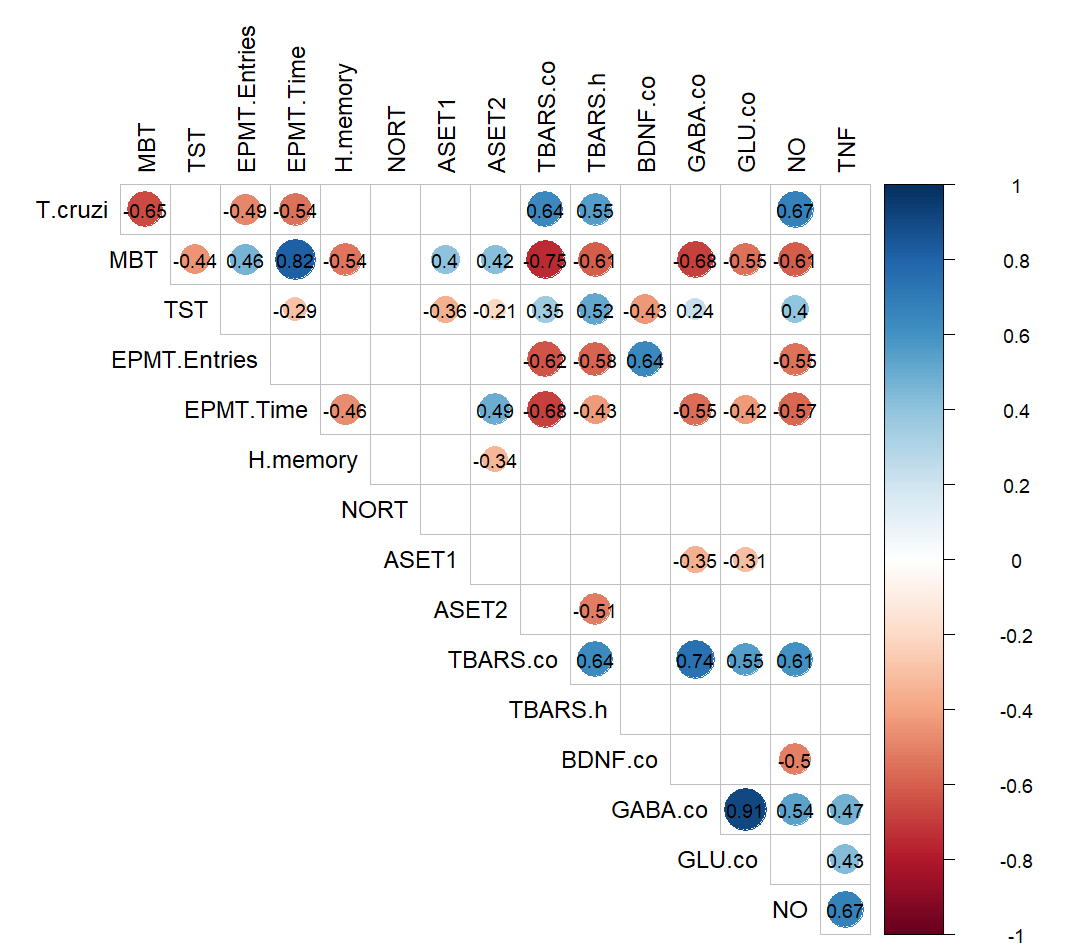

Supplement: S8 Fig — Spearman correlation significance test was applied. (TIF) [file pone.0334708.s008.tif]
