# Supplementary material for: Combined benznidazole and pentoxifylline therapy improves behavioral and cognitive changes in association with the regulation of systemic inflammatory profile in chronic experimental Chagas disease
Source: PLoS One. 2025 Nov 14;20(11):e0334708. doi: 10.1371/journal.pone.0334708 (PMC12617855; doi:10.1371/journal.pone.0334708)
Supplement: S1 Table — (DOCX) [file pone.0334708.s009.docx]

**S1 Table.** Data used to build graphs and figures.

| **Fig 1A** | Parasitemia parasites (x10^3^)/mL | | | | | | |
| --- | --- | --- | --- | --- | --- | --- | --- |
| **Days post-infection** | **7** | **14** | **28** | **42** | **90** | **120** | **150** |
| Mean ± SD | 0 ± 0 | 3,80 ± 4,86 | 62,22 ± 49,09 | 152,09 ± 107,14 | 12,44 ± 17,04 | 2,07 ± 3,11 | 0,69 ± 2.01 |

| **Fig 1B** | **Experimental design** |
| --- | --- |

| **Fig 1C** | Parasitemia parasites (x10^3^)/mL | | | | |
| --- | --- | --- | --- | --- | --- |
|  | **NI** | **Veh** | **PTX** | **Bz** | **Bz+PTX** |
| Mean ± SD | 0 ± 0 | 71,11 ± 22,00 | 67,30 ± 47,10 | 18,98 ± 33, 32 | 11,57 ± 14,58 |

| **Fig 1D** | Parasite equivalents / mg tissue | | | | |
| --- | --- | --- | --- | --- | --- |
| **Individual data** | **cortex** | | | | |
|  | **NI** | **Veh** | **PTX** | **Bz** | **Bz+PTX** |
|  | 0 | 16.24 | 0.01 | 1.36 | 1.13 |
|  | 0 | 20.58 | 2.14 | 0.41 | 0.44 |
|  | 0 | 17.22 | 0.12 | 0.3 | 0.09 |
| Mean ± SD | 0 ± 0 | 18.01 ± 2.28 | 0.76 ± 1.19 | 0.69 ± 0.58 | 0.55 ± 0.53 |
|  |  |  |  |  |  |
|  | **hippocampus** | | | | |
|  | **NI** | **Veh** | **PTX** | **Bz** | **Bz+PTX** |
|  | 0 | 8.11 | 0.01 | 2.81 | 0.56 |
|  | 0 | 9.33 | 0.83 | 0.18 | 0.88 |
|  | 0 | 7.28 | 0 | 0.43 | 0.58 |
| Mean ± SD | 0 ± 0 | 8.24 ± 1.03 | 0.28 ± 0.48 | 1.14 ± 1.45 | 0.67 ± 0.18 |

| Sample | Parasite Load  (Par. Eq.) | Tissue mass  (mg) | Par. Eq./  mg of tissue |  | Sample | Parasite Load  (Par. Eq.) | Tissue mass  (mg) | Par. Eq./  mg of tissue |
| --- | --- | --- | --- | --- | --- | --- | --- | --- |
| NI1C | 0 | 6.67 | 0.00 |  | NI1H | 0 | 5.547 | 0.00 |
| NI2C | 0 | 10.01 | 0.00 |  | NI2H | 0 | 3.644 | 0.00 |
| NI3C | 0 | 12.76 | 0.00 |  | NI3H | 0 | 16.825 | 0.00 |
| Veh1C | 247.18 | 15.22 | 16.24 |  | Veh1H | 199.54 | 24.598 | 8.11 |
| Veh2C | 578.13 | 28.10 | 20.58 |  | Veh2H | 151.53 | 16.234 | 9.33 |
| Veh3C | 149.04 | 8.66 | 17.22 |  | Veh3H | 183.28 | 25.16 | 7.28 |
| Bz1C | 0.07 | 13.49 | 0.01 |  | Bz1H | 0.25 | 24.885 | 0.01 |
| Bz2C | 59.28 | 27.74 | 2.14 |  | Bz2H | 19.44 | 23.39 | 0.83 |
| Bz3C | 5.45 | 46.69 | 0.12 |  | Bz3H | 0.02 | 54.219 | 0.00 |
| PTX1C | 109.12 | 80.22 | 1.36 |  | PTX1H | 19.99 | 7.115 | 2.81 |
| PTX2C | 9.44 | 22.82 | 0.41 |  | PTX2H | 3.63 | 19.686 | 0.18 |
| PTX3C | 43.6 | 144.24 | 0.30 |  | PTX3H | 8.43 | 19.619 | 0.43 |
| Bz+PTX1C | 110.23 | 97.43 | 1.13 |  | Bz+PTX1H | 3.33 | 5.952 | 0.56 |
| Bz+PTX2C | 47.5 | 107.42 | 0.44 |  | Bz+PTX2H | 2.17 | 2.468 | 0.88 |
| Bz+PTX3C | 16.56 | 194.15 | 0.09 |  | Bz+PTX3H | 15.27 | 26.41 | 0.58 |

Hippocampus – pairwise comparisons using Dunn´s many-to-one test

| Group | H1 | Control | Median Diff | Significant? | Adjusted P Value |
| --- | --- | --- | --- | --- | --- |
| PTX | < | Veh | -8.1 | Yes | 0.014 |
| Bz |  |  | -7.68 | No | 0.054 |
| Bz + PTX |  |  | -7.53 | No | 0.056 |
| PTX | > | NI | 0.01 | No | 0.125 |
| Bz |  |  | 0.43 | Yes | 0.044 |
| Bz + PTX |  |  | 0.58 | Yes | 0.027 |

Cortex – pairwise comparisons using Dunn´s many-to-one test

| Group | H1 | Control | Median Diff | Significant? | Adjusted P Value |
| --- | --- | --- | --- | --- | --- |
| PTX | < | Veh | -17.1 | Yes | 0.035 |
| Bz |  |  | -16.81 | Yes | 0.042 |
| Bz + PTX |  |  | -16.78 | Yes | 0.042 |
| PTX | > | NI | 0.12 | Yes | 0.040 |
| Bz |  |  | 0.41 | Yes | 0.034 |
| Bz + PTX |  |  | 0.44 | Yes | 0.040 |

| **Fig 2A** | **MBT** | | | | |
| --- | --- | --- | --- | --- | --- |
|  | Number of buried spheres (Mean ± SD) | | | | |
| **minutes** | **NI** | **Veh** | **PTX** | **Bz** | **Bz+PTX** |
| 5 | 14.13 ± 5.34 | 3.44 ± 4.39 | 13.91 ± 4.89 | 9.5 ± 4.47 | 10.6 ± 4.99 |
| 10 | 18.13 ± 3.33 | 5.81 ± 9.09 | 17 ± 4.58 | 13.3 ± 4.03 | 15.6 ± 3.56 |
| 15 | 20 ± 0 | 6.75 ± 7.29 | 18.091 ± 3.58 | 15.4 ± 4.99 | 19.2 ± 1.75 |
| 20 | 20 ± 0 | 6.75 ± 7.29 | 18.091 ± 3.53 | 15.7 ± 5.05 | 19.7 ± 0.95 |
| 25 | 20 ± 0 | 7.12 ± 7.67 | 18.091 ± 3.53 | 15.7 ± 5.05 | 19.7 ± 0.95 |
| 30 | 20 ± 0 | 7.12 ± 7.67 | 18.091 ± 3.53 | 15.7 ± 5.05 | 19.7 ± 0.95 |

| **Fig 2B** | **EPMT** | | | | |
| --- | --- | --- | --- | --- | --- |
|  | Number of entries | | | | |
|  | **NI** | **Veh** | **PTX** | **Bz** | **Bz+PTX** |
| Mean ± SD | 5.93 ± 1.75 | 1.2 ± 0.63 | 2.2 ± 0.79 | 2.36 ± 1.21 | 2 ± 0.81 |
|  |  |  |  |  |  |
|  | Time (s) | | | | |
|  | **NI** | **Veh** | **PTX** | **Bz** | **Bz+PTX** |
| Mean ± SD | 46.94 ± 7.91 | 13.56 ± 6.62 | 34.39 ± 16.65 | 25.7 ± 3.13 | 43.5 ± 5.50 |

| **Fig 2C** | **TST** | | | | |
| --- | --- | --- | --- | --- | --- |
|  | Immobility (s) | | | | |
|  | **NI** | **Veh** | **PTX** | **Bz** | **Bz+PTX** |
| Mean ± SD | 30.6 ± 8.11 | 117.7 ± 16.44 | 35.25 ± 10.05 | 27.58 ± 15.56 | 33.23 ± 18.07 |

| **Fig 3A** | **NORT** |  |  |  |  |
| --- | --- | --- | --- | --- | --- |
|  | Discrimination index | | | | |
|  | **NI** | **Veh** | **PTX** | **Bz** | **Bz+PTX** |
| Mean ± SD | 0.61 ± 0.06 | 0.36 ± 0.08 | 0.56 ± 0.10 | 0.60 ± 0.59 | 0.56 ± 0.03 |

| **Fig 3B** | **OFT2** |  |  |  |  |
| --- | --- | --- | --- | --- | --- |
|  | Discrimination index | | | | |
|  | **NI** | **Veh** | **PTX** | **Bz** | **Bz+PTX** |
| Mean ± SD | 0.33 ± 0.04 | 0.47 ± 0.15 | 0.37 ± 0.17 | 0.32 ± 0.08 | 0.39 ± 0.09 |

| **Fig 3C** | **Aversive shock evoked test** | | | | | | | | | |
| --- | --- | --- | --- | --- | --- | --- | --- | --- | --- | --- |
|  | Latency (s) | | | | | | | | |  |
|  | **NI** | | **Veh** | | **PTX** | | **Bz** | | **Bz+PTX** | |
| Mean ± SD | 31.24 ± 5.96 | 114.91 ± 10.66 | **23.86 ± 9.28** | **89.07 ± 28.90** | 33.51 ± 10.51 | 114.85 ± 10.25 | **31.31 ± 7.39** | **111.64 ± 12.73** | 33.19 ± 7.21 | 114.36 ± 11.24 |

| **Fig 4A** | **TBARS** OD (450 nm) | | | | |
| --- | --- | --- | --- | --- | --- |
| **cortex** |  |  | |  |  |
|  | **NI** | **Veh** | **PTX** | **Bz** | **Bz+PTX** |
| Mean ± SD | 0.23 ± 0.05 | 0.45 ± 0.05 | 0.37 ± 0.02 | 0.33 ± 0.08 | 0.35 ± 0.06 |

| **Fig 4B** | **TBARS** OD (450 nm) | | | | |
| --- | --- | --- | --- | --- | --- |
| **Hippocampus** |  |  | |  |  |
|  | **NI** | **Veh** | **PTX** | **Bz** | **Bz+PTX** |
| Mean ± SD | 0.27 ± 0.03 | 0.51 ± 0.07 | 0.37 ± 0.06 | 0.44 ± 0.22 | 0.36 ± 0.09 |

| **Fig 5A** | **GABA** (mM) | | | | |
| --- | --- | --- | --- | --- | --- |
| **cortex** |  |  |  |  |  |
|  | **NI** | **Veh** | **PTX** | **Bz** | **Bz+PTX** |
| Mean ± SD | 0.40 ± 0.16 | 2.00 ± 0.20 | 0.48 ± 0.30 | 1.62 ± 0.31 | 0.63 ± 0.38 |

| **Fig 5B** |  |  |  |  |  |
| --- | --- | --- | --- | --- | --- |
| **cortex** | **Glutamate** (mM) | | | | |
|  | **NI** | **Veh** | **PTX** | **Bz** | **Bz+PTX** |
| Mean ± SD | 0.72 ± 0.33 | 1.52 ± 0.19 | 0.59 ± 0.37 | 1.52 ± 0.25 | 0.83 ± 0.46 |

| **Fig 5C** | **BDNF mRNA** (relative expression) | | | | |
| --- | --- | --- | --- | --- | --- |
| **cortex** |  |  | |  |  |
|  | **NI** | **Veh** | **PTX** | **Bz** | **Bz+PTX** |
| Mean ± SD | 1.00 ± 0.0 | 0.55 ± 0.18 | 0.77 ± 0.07 | 0.91 ± 0.31 | 0.69 ± 0.16 |

| **Fig 6A** | **NO (µM)** | | | | | | |
| --- | --- | --- | --- | --- | --- | --- | --- |
| **Days post-infection** | **NI** | **14** | **28** | **42** | **90** | **120** | **150** |
| Mean ± SD | 50.89 ± 19.64 | 72.12 ± 25.67 | 128.29 ± 51.90 | 216.83 ± 87.21 | 90.28 ± 22.78 | 134.17 ± 67.28 | 169.55 ± 72.33 |

| **Fig 6B** | **NO_x_ (µM)** | | | | |
| --- | --- | --- | --- | --- | --- |
|  | **NI** | **Veh** | **PTX** | **Bz** | **Bz+PTX** |
| Mean ± SD | 39.74 ± 10.31 | 374.80 ± 175.96 | 320.98 ± 200.63 | 143.31 ± 31.54 | 88.44 ± 30.68 |

| **Fig 6C** | **TNF (pg/mL)** | | | | | | |
| --- | --- | --- | --- | --- | --- | --- | --- |
|  | Pre-therapy (120 dpi) | | Post-therapy (150 dpi) | | | | |
|  | **NI** | **Veh** | **NI** | **Veh** | **PTX** | **Bz** | **Bz+PTX** |
| Mean ± SD | 7.80 ± 3.72 | 50.06 ± 38.65 | 9.65 ± 2.32 | 118.62 ± 74.62 | 58.27 ± 25.93 | 61.41 ± 69.01 | 61.07 ± 43.03 |

| **Fold increase** | | | |  | **Fold increase** | | | | | | |
| --- | --- | --- | --- | --- | --- | --- | --- | --- | --- | --- | --- |
| **Fig 7A** | **Vehicle** |  |  |  |  |  |  |  |  |  |  |
|  | **Blood** |  |  |  | **Heart** |  |  |  |  |  |  |
|  | **TNF** | **NO** | **IL-6** |  | **TNF** | **IFNγ** | **IL-10** | **IL-12a** | **IL-12b** | **IL-2** | **IL-6** |
|  | 12.29 | 9.43 | 1.42 |  | 24.35 | 126.51 | 16.57 | 2.12 | 44.35 | 18.20 | 3.88 |

| **Fig 7B** | **Bz** |  |  |  |  |  |  |  |  |  |  |
| --- | --- | --- | --- | --- | --- | --- | --- | --- | --- | --- | --- |
|  | **Blood** |  |  |  | **Heart** |  |  |  |  |  |  |
|  | **TNF** | **NO** | **IL-6** |  | **TNF** | **IFNγ** | **IL-10** | **IL-12a** | **IL-12b** | **IL-2** | **IL-6** |
|  | 6.37 | 3.61 | 1.31 |  | 15.38 | 11.16 | 29.69 | 0.93 | 9.21 | 2.22 | 0.23 |

| **Fig 7C** | **Bz+PTX** |  |  |  |  |  |  |  |  |  |  |
| --- | --- | --- | --- | --- | --- | --- | --- | --- | --- | --- | --- |
|  | **Blood** |  |  |  | **Heart** |  |  |  |  |  |  |
|  | **TNF** | **NO** | **IL-6** |  | **TNF** | **IFNγ** | **IL-10** | **IL-12a** | **IL-12b** | **IL-2** | **IL-6** |
|  | 6.33 | 2.22 | 0.81 |  | 16.08 | 8.71 | 4.14 | 1.47 | 5.24 | 2.68 | 0.17 |

| **Figure 9** | |  |  |  |  |  |  |  |  |  |  |  |  |  |  |  |
| --- | --- | --- | --- | --- | --- | --- | --- | --- | --- | --- | --- | --- | --- | --- | --- | --- |
| **PCA** | | | | | | | | | | | | | | | | |
| **Data** | | | | | | | | | | | | | | | | |
| **Group** | *T.cruzi* | MBT | TST | EPMT-Entries | EPMT-Time | Habituation  memory | NORT | ASET1 | ASET2 | TBARS-co | TBARS-h | BDNF-co | GABA-co | GLU-co | NO | TNF |
| **NI** | 0 | 20 | 35 | 6 | 48 | 0.35 | 0.58 | 35.21 | 120 | 0.22 | 0.28 | 1 | 0.22 | 0.5 | 24.78 | 10.12 |
| **NI** | 0 | 20 | 27 | 4 | 42.3 | 0.31 | 0.63 | 22.52 | 120 | 0.16 | 0.23 | 1 | 0.31 | 1 | 48.15 | 13.06 |
| **NI** | 0 | 20 | 38 | 5 | 25 | 0.281 | 0.54 | 31.71 | 120 | 0.29 | 0.25 | 1 | 0.53 | 0.37 | 44.20 | 9.93 |
| **NI** | 0 | 20 | 35 | 8 | 54.6 | 0.35 | 0.56 | 30.32 | 120 | 0.19 | 0.26 | 1 | 0.55 | 1.01 | 41.82 | 9.69 |
| **Veh** | 49,8 | 0 | 127 | 1 | 11.33 | 0.62 | 0.73 | 10.7 | 42.13 | 0.43 | 0.54 | 0.51 | 2.22 | 1.5 | 171.26 | 60.44 |
| **Veh** | 55,99 | 5 | 98 | 1 | 19.33 | 0.25 | 0.42 | 11.43 | 120 | 0.52 | 0.53 | 0.44 | 1.91 | 1.62 | 603.91 | 119.36 |
| **Veh** | 105,77 | 0 | 102 | 1 | 12.6 | 0.53 | 0.53 | 22.75 | 120 | 0.46 | 0.59 | 0.44 | 1.86 | 1.52 | 341.86 | 21.95 |
| **Veh** | 68,44 | 10 | 119 | 2 | 13.3 | 0.47 | 0.37 | 32.21 | 78 | 0.47 | 0.40 | 0.82 | 1.99 | 1.74 | 498.53 | 255.68 |
| **PTX** | 112,00 | 20 | 40 | 2 | 50 | 0.13 | 0.61 | 25.2 | 120 | 0.36 | 0.35 | 0.83 | 0.71 | 0.88 | 643.71 | 90.65 |
| **PTX** | 49,78 | 16 | 35 | 3 | 19.7 | 0.61 | 0.62 | 47.8 | 98.7 | 0.36 | 0.38 | 0.78 | 0.6 | 0.73 | 362.85 | 76.28 |
| **PTX** | 6,22 | 20 | 29 | 1 | 34 | 0.21 | 0.63 | 45.9 | 94.98 | 0.37 | 0.41 | 0.69 | 0.14 | 0.18 | 151.73 | 57.72 |
| **PTX** | 87,11 | 13 | 28 | 3 | 19 | 0.46 | 0.66 | 27 | 120 | 0.33 | 0.27 | 0.77 | 0.48 | 0.59 | 162.58 | 58 |
| **Bz** | 6,22 | 7 | 33 | 3 | 27 | 0.42 | 0.70 | 19.87 | 120 | 0.39 | 0.29 | 1.06 | 1.98 | 1.74 | 181.12 | 153.96 |
| **Bz** | 74,66 | 11 | 0 | 2 | 22 | 0.36 | 0.61 | 32.2 | 96.89 | 0.42 | 0.73 | 1.16 | 1.45 | 1.24 | 92.66 | 85.63 |
| **Bz** | 0 | 20 | 26 | 3 | 24 | 0.17 | 0.57 | 31.4 | 120 | 0.28 | 0.28 | 0.57 | 1.43 | 1.58 | 169.18 | 176.75 |
| **Bz** | 0 | 20 | 16 | 5 | 28 | 0.314 | 0.64 | 40.9 | 120 | 0.39 | 0.28 | 0.91 | 1.62 | 1.52 | 106.49 | 11.17 |
| **Bz+PTX** | 31,11 | 20 | 12 | 2 | 35 | 0.39 | 0.56 | 29.98 | 120 | 0.40 | 0.24 | 0.88 | 0.96 | 1.11 | 83.70 | 12.94 |
| **Bz+PTX** | 24,89 | 20 | 48 | 3 | 36 | 0.47 | 0.57 | 28.2 | 120 | 0.29 | 0.40 | 0.58 | 0.22 | 0.3 | 126.85 | 67.49 |
| **Bz+PTX** | 0 | 20 | 0 | 1 | 44 | 0.42 | 0.56 | 29.64 | 120 | 0.28 | 0.25 | 0.63 | 0.7 | 1.08 | 108.58 | 54.93 |
| **Bz+PTX** | 0 | 20 | 42 | 2 | 51 | 0.29 | 0.62 | 26.89 | 120 | 0.35 | 0.43 | 0.69 | 0.63 | 0.83 | 47.16 | 128.97 |

| **Figure 9** | |  |  |  |  |  |  |  |  |  |  |  |  |  |  |  |
| --- | --- | --- | --- | --- | --- | --- | --- | --- | --- | --- | --- | --- | --- | --- | --- | --- |
| **PCA** | | | | | | | | | | | | | | | | |
| **Transfdata** | | | | | | | | | | | | | | | | |
| **Group** | *T.cruzi* | MBT | TST | EPMT-Entries | EPMT-Time | Habituation  memory | NORT | ASET1 | ASET2 | TBARS-co | TBARS-h | BDNF-co | GABA-co | GLU-co | NO | TNF |
| **NI** | 0 | 20 | 35 | 2.45 | 6.93 | 0.36 | 0.58 | 35.21 | 2.08 | 0.22 | 0.53 | 1.00 | 0.47 | 0.50 | 4.98 | 3.18 |
| **NI** | 0 | 20 | 27 | 2.00 | 6.50 | 0.31 | 0.63 | 22.52 | 2.08 | 0.16 | 0.48 | 1.00 | 0.56 | 1.00 | 6.94 | 13.06 |
| **NI** | 0 | 20 | 38 | 2.24 | 5.00 | 0.28 | 0.54 | 31.71 | 2.08 | 0.30 | 0.50 | 1.00 | 0.73 | 0.37 | 6.65 | 9.93 |
| **NI** | 0 | 20 | 35 | 2.83 | 7.39 | 0.35 | 0.56 | 30.32 | 2.08 | 0.19 | 0.51 | 1.00 | 0.74 | 1.01 | 6.47 | 9.69 |
| **Veh** | 49,80 | 0 | 127 | 1.00 | 3.37 | 0.62 | 0.72 | 10.70 | 1.62 | 0.43 | 0.74 | 0.51 | 1.49 | 1.50 | 13.09 | 60.44 |
| **Veh** | 55,99 | 5 | 98 | 1.00 | 4.40 | 0.25 | 0.42 | 11.43 | 2.08 | 0.52 | 0.73 | 0.44 | 1.38 | 1.62 | 24.57 | 119.36 |
| **Veh** | 105,77 | 0 | 102 | 1.00 | 3.55 | 0.53 | 0.53 | 22.75 | 2.08 | 0.46 | 0.77 | 0.44 | 1.36 | 1.52 | 18.49 | 21.95 |
| **Veh** | 68,44 | 10 | 119 | 1.41 | 3.65 | 0.47 | 0.37 | 32.21 | 1.89 | 0.47 | 0.63 | 0.82 | 1.41 | 1.74 | 22.33 | 255.68 |
| **PTX** | 112,00 | 20 | 40 | 1.41 | 7.07 | 0.13 | 0.61 | 25.20 | 2.08 | 0.36 | 0.59 | 0.83 | 0.84 | 0.88 | 25.37 | 90.65 |
| **PTX** | 49,78 | 16 | 35 | 1.73 | 4.44 | 0.61 | 0.62 | 47.80 | 1.99 | 0.37 | 0.62 | 0.78 | 0.77 | 0.73 | 19.05 | 76.28 |
| **PTX** | 6,22 | 20 | 29 | 1.00 | 5.83 | 0.21 | 0.63 | 45.90 | 1.98 | 0.38 | 0.64 | 0.69 | 0.37 | 0.18 | 12.32 | 57.72 |
| **PTX** | 87,11 | 13 | 28 | 1.73 | 4.36 | 0.46 | 0.66 | 27.00 | 2.08 | 0.33 | 0.52 | 0.77 | 0.70 | 0.60 | 12.75 | 58.00 |
| **Bz** | 6,22 | 7 | 33 | 1.73 | 5.20 | 0.42 | 0.70 | 19.87 | 2.08 | 0.40 | 0.54 | 1.01 | 1.41 | 1.74 | 13.46 | 153.96 |
| **Bz** | 74,66 | 11 | 0 | 1.41 | 4.69 | 0.36 | 0.61 | 32.20 | 1.99 | 0.42 | 0.85 | 1.16 | 1.20 | 1.24 | 9.63 | 85.63 |
| **Bz** | 0 | 20 | 26 | 1.73 | 4.90 | 0.17 | 0.57 | 31.40 | 2.08 | 0.28 | 0.53 | 0.57 | 1.20 | 1.58 | 13.01 | 176.75 |
| **Bz** | 0 | 20 | 16 | 2.24 | 5.29 | 0.30 | 0.64 | 40.90 | 2.08 | 0.39 | 0.53 | 0.91 | 1.27 | 1.52 | 10.32 | 11.17 |
| **Bz+PTX** | 31,11 | 20 | 12 | 1.41 | 5.92 | 0.39 | 0.56 | 29.98 | 2.08 | 0.41 | 0.49 | 0.89 | 0.98 | 1.11 | 9.15 | 12.94 |
| **Bz+PTX** | 24,89 | 20 | 48 | 1.73 | 6.00 | 0.47 | 0.57 | 28.20 | 2.08 | 0.29 | 0.64 | 0.58 | 0.47 | 0.30 | 11.26 | 67.49 |
| **Bz+PTX** | 0 | 20 | 0 | 1.00 | 6.63 | 0.42 | 0.56 | 29.64 | 2.08 | 0.28 | 0.51 | 0.63 | 0.84 | 1.08 | 10.42 | 54.93 |
| **Bz+PTX** | 0 | 20 | 42 | 1.41 | 7.14 | 0.30 | 0.62 | 26.89 | 2.08 | 0.35 | 0.66 | 0.70 | 0.79 | 0.83 | 6.87 | 128.97 |

| Data used for Variance-stabilizing transformation. |  | All data [y = √x] , except for ASET2 [y= log_10_ (x)] |  |
| --- | --- | --- | --- |

| **S2A Fig** | **Body weight (g)** | | | | | | |
| --- | --- | --- | --- | --- | --- | --- | --- |
|  | Pre-therapy (120 dpi) | | Post-therapy (150 dpi) | | | | |
|  | **NI** | **Veh** | **NI** | **Veh** | **PTX** | **Bz** | **Bz+PTX** |
| Mean ± SD | 22.70 ± 0.70 | 20.62 ± 2.99 | 23.23 ± 1.31 | 20.58 ± 1.35 | 22.53 ± 2.05 | 19.60 ± 2.02 | 18.90 ± 1.67 |

| **S2B Fig** | **Spleen relative weight (mg/g)** | | | | | | |
| --- | --- | --- | --- | --- | --- | --- | --- |
|  |  |  |  |  |  |  |  |
|  | Pre-therapy (120 dpi) | | Post-therapy (150 dpi) | | | | |
|  | **NI** | **Veh** | **NI** | **Veh** | **PTX** | **Bz** | **Bz+PTX** |
| Mean ± SD | 4.15 ± 0.10 | 21.15 ± 4.58 | 3.01 ± 0.28 | 24.40 ± 6.35 | 15.99 ± 7.89 | 12.13 ± 1.78 | 10.32 ± 1.09 |
