# Supplementary material for: Combined benznidazole and pentoxifylline therapy improves behavioral and cognitive changes in association with the regulation of systemic inflammatory profile in chronic experimental Chagas disease
Source: PLoS One. 2025 Nov 14;20(11):e0334708. doi: 10.1371/journal.pone.0334708 (PMC12617855; doi:10.1371/journal.pone.0334708)
Supplement: S2 Table — (DOCX) [file pone.0334708.s010.docx]

**S2 Table.** Pre-therapy and pos-therapy serum concentrations of cytokines and NO.

| **Group** | **TNF**  **(pg/mL)** | **IFNγ**  **(pg/mL)** | **IL-6**  **(pg/mL)** | **IL-10**  **(pg/mL)** | **NO**  **(µM)** |
| --- | --- | --- | --- | --- | --- |
| **Pre-Therapy**  **(120 dpi)** |  |  |  |  |  |
| NI | 7.80 ± 3.72 | 1.11 ± 0.57 | NT | 8.39 ± 3.15 | 50.9 ± 19.6 |
| *T. cruzi* | 50.07 ± 38.65^**^ | 22.80 ± 21.08^*^ | NT | 12.81 ± 3.97 | 134.2 ± 67.4^*^ |
|  |  |  |  |  |  |
| **Pos-Therapy**  **(150 dpi)** |  |  |  |  |  |
| NI | 11.15 ± 3.43 | 0.48 ± 0.29 | 71.1 ± 10.2 | 9.99 ± 2.61 | 39.7 ± 10.3 |
| *T. cruzi* |  |  |  |  |  |
| Veh | 132.4 ± 68.7^**^ | 40.04 ± 33.16^*^ | 101.2 ± 63.4 | 13.78 ± 4.02^*^ | 374.8 ± 176^**^ |
| PTX | 58.28 ± 25.93^*,#^ | 61.61 ± 31.82^*^ | 24.8 ± 25.18 | 16.33 ± 4.25 | 321 ± 200^*^ |
| Bz | 87.2 ± 78.18^*^ | 27.01 ± 19.62^*^ | 94.4 ± 50.9 | 11.92 ± 7.27 | 143 ± 31.5^*,#^ |
| Bz+PTX | 61.07 ± 43.3^*^ | 43.42 ± 11.26^*^ | 23.2 ± 23.89 | 11.45 ± 2.80 | 88.4 ± 30.7^*,##^ |

NT, not tested. Data are shown as the means ± SD. ^*^, *p* < 0.05, ^**^, *p* < 0.01 and ^***^, *p* < 0.001, *T. cruzi*-infected compared with NI controls. ^#^, *p* < 0.05 and ^##^, *p* < 0.01, treatments compared with Veh-treated *T. cruzi*-infected mice.
