# Supplementary material for: Combined benznidazole and pentoxifylline therapy improves behavioral and cognitive changes in association with the regulation of systemic inflammatory profile in chronic experimental Chagas disease
Source: PLoS One. 2025 Nov 14;20(11):e0334708. doi: 10.1371/journal.pone.0334708 (PMC12617855; doi:10.1371/journal.pone.0334708)
Supplement: S3 Table — (DOCX) [file pone.0334708.s011.docx]

### S3 Table. List of up- or downregulated microRNAs (1.3-fold change) in the vehicle-treated group.

| **Name** | **Accession number** | **Fold Change** |
| --- | --- | --- |
| mmu-miR-182-5p | MIMAT0000211 | 15.853 |
| mmu-miR-146a-5p | MIMAT0000158 | 4.961 |
| mmu-miR-146b-5p | MIMAT0003475 | 4.070 |
| mmu-miR-146b-5p | MIMAT0005845 | 3.992 |
| mmu-miR-155-5p | MIMAT0000165 | 3.088 |
| mmu-let-7d-3p | MIMAT0000384 | 1.968 |
| mmu-miR-133a-5p | MIMAT0003473 | 1.919 |
| mmu-miR-132-3p | MIMAT0000144 | 1.860 |
| mmu-miR-223-3p | MIMAT0000665 | 1.785 |
| mmu-miR-148b-3p | MIMAT0000580 | 1.663 |
| mmu-miR-21-5p | MIMAT0000530 | 1.581 |
| rno-miR-29c-5p | MIMAT0003154 | 0.697 |
| hsa-miR-93-3p | MIMAT0004509 | 0.689 |
| mmu-miR-335-3p | MIMAT0004704 | 0.683 |
| hsa-miR-29a-5p | MIMAT0004503 | 0.677 |
| mmu-miR-133b-3p | MIMAT0000769 | 0.674 |
| rno-miR-7a-1-3p | MIMAT0000607 | 0.583 |
| mmu-miR-143-3p | MIMAT0000247 | 0.553 |
| hsa-miR-223-3p | MIMAT0000280 | 0.541 |
| mmu-miR-7a-1-3p | MIMAT0004670 | 0.510 |
| mmu-miR-335-5p | MIMAT0000766 | 0.449 |
| mmu-miR-133a-3p | MIMAT0000145 | 0.385 |
| mmu-miR-9-5p | MIMAT0000142 | 0.297 |
| rno-miR-99a-3p | MIMAT0004724 | 0.188 |
