# Supplementary material for: Combined benznidazole and pentoxifylline therapy improves behavioral and cognitive changes in association with the regulation of systemic inflammatory profile in chronic experimental Chagas disease
Source: PLoS One. 2025 Nov 14;20(11):e0334708. doi: 10.1371/journal.pone.0334708 (PMC12617855; doi:10.1371/journal.pone.0334708)
Supplement: S4 Table — (DOCX) [file pone.0334708.s012.docx]

### S4 Table. List of up- or downregulated microRNAs restored with Bz treatment.

| **Name** | **Accession number** | **Fold Change** |
| --- | --- | --- |
| mmu-miR-146b-5p | MIMAT0003475 | 4.070 |
| rno-miR-146b-5p | MIMAT0005595 | 1.967 |
| mmu-miR-223-3p | MIMAT0000665 | 1.785 |
| mmu-miR-133b-3p | MIMAT0000769 | 0.674 |
| rno-miR-7a-1-3p | MIMAT0000607 | 0.583 |
| hsa-miR-223-3p | MIMAT0000280 | 0.541 |
| mmu-miR-7a-1-3p | MIMAT0004670 | 0.510 |
| mmu-miR-133a-3p | MIMAT0000145 | 0.385 |
| mmu-miR-9-5p | MIMAT0000142 | 0.297 |
