# Supplementary material for: Combined benznidazole and pentoxifylline therapy improves behavioral and cognitive changes in association with the regulation of systemic inflammatory profile in chronic experimental Chagas disease
Source: PLoS One. 2025 Nov 14;20(11):e0334708. doi: 10.1371/journal.pone.0334708 (PMC12617855; doi:10.1371/journal.pone.0334708)
Supplement: S5 Table — (DOCX) [file pone.0334708.s013.docx]

**S5 Table.** List of up- or downregulated microRNAs restored with Bz+PTX treatment.

| **Name** | **Accesion number** | **Fold Change** |
| --- | --- | --- |
| mmu-miR-146b-5p | MIMAT0003475 | 4.070 |
| rno-miR-146b-5p | MIMAT0005595 | 1.967 |
| mmu-miR-132-3p | MIMAT0000144 | 1.860 |
| mmu-miR-148b-3p | MIMAT0000580 | 1.663 |
| mmu-miR-21-5p | MIMAT0000530 | 1.581 |
| mmu-miR-335-3p | MIMAT0004704 | 0.683 |
| mmu-miR-133b-3p | MIMAT0000769 | 0.674 |
| hsa-miR-223-3p | MIMAT0000280 | 0.541 |
| mmu-miR-7a-1-3p | MIMAT0004670 | 0.510 |
