# Supplementary material for: Combined benznidazole and pentoxifylline therapy improves behavioral and cognitive changes in association with the regulation of systemic inflammatory profile in chronic experimental Chagas disease
Source: PLoS One. 2025 Nov 14;20(11):e0334708. doi: 10.1371/journal.pone.0334708 (PMC12617855; doi:10.1371/journal.pone.0334708)
Supplement: S6 Table — (DOCX) [file pone.0334708.s014.docx]

**Table S6. Values of observed correlations between behavioral, cognitive and neurochemical variables.**

|  | **MBT** | **TST** | **EPMT.Entries** | | | **EPMT.Time** | | | **H.memory** | | **NORT** | | **ASET1** | | | **ASET2** | | **TBARS.co** | | | **TBARS.h** | | **BDNF.co** | | **GABA.co** | | **GLU.co** | | | **NO** | **TNF** |  |
| --- | --- | --- | --- | --- | --- | --- | --- | --- | --- | --- | --- | --- | --- | --- | --- | --- | --- | --- | --- | --- | --- | --- | --- | --- | --- | --- | --- | --- | --- | --- | --- | --- |
| ***T. cruzi*** | **-0.65** | 0.36 | **-0.49** | | | **-0.54** | | | 0.34 | | -0.06 | | -0.22 | | | -0.35 | | **0.64** | | | **0.55** | | -0.23 | | 0.34 | | 0.14 | | | **0.67** | 0.34 |  |
|  | **MBT** | **-0.44** | **0.46** | | | **0.82** | | | **-0.54** | | -0.02 | | **0.4** | | | **0.42** | | **-0.75** | | | **-0.61** | | 0.27 | | **-0.68** | | **-0.55** | | | **-0.61** | -0.4 |  |
|  |  | **TST** | -0.22 | | | **-0.29** | | | 0.23 | | -0.22 | | **-0.36** | | | **-0.21** | | **0.35** | | | **0.52** | | **-0.43** | | **0.24** | | 0.03 | | | **0.4** | 0.25 |  |
|  |  |  | | **EPMT.Entries** | | 0.37 | | | -0.14 | | 0.17 | | 0.34 | | | 0.39 | | **-0.62** | | | **-0.58** | | **0.64** | | -0.42 | | -0.24 | | | **-0.55** | -0.42 |  |
|  |  |  | | |  | | **EPMT.Time** | | **-0.46** | | 0.02 | | 0.1 | | | **0.49** | | **-0.68** | | | **-0.43** | | 0.38 | | **-0.55** | | **-0.42** | | | **-0.57** | -0.32 |  |
|  |  |  | | |  | | |  | **H.memory** | | 0.06 | | -0.1 | | | **-0.34** | | 0.24 | | | 0.2 | | -0.17 | | 0.22 | | 0.09 | | | 0.17 | -0.02 |  |
|  |  |  | | |  | | |  | |  | | **NORT** | -0.11 | | | -0.17 | | -0.12 | | | 0 | | 0.21 | | -0.1 | | -0.15 | | | -0.03 | 0.03 |  |
|  |  |  | | |  | | |  | |  | |  | | **ASET1** | | -0.29 | | -0.15 | | | -0.14 | | 0.3 | | **-0.35** | | **-0.31** | | | -0.27 | -0.23 |  |
|  |  |  | | |  | | |  | |  | |  | |  | | **ASET2** | | -0.47 | | | -0.51 | | 0.08 | | -0.25 | | -0.06 | | | -0.3 | -0.29 |  |
|  |  |  | | |  | | |  | |  | |  | | |  | |  | | **TBARS.co** | | **0.64** | | -0.27 | | **0.74** | | **0.55** | | | **0.61** | 0.42 |  |
|  |  |  | | |  | | |  | |  | |  | | |  | |  | | |  | **TBARS.h** | | -0.42 | | 0.37 | | 0.17 | | | 0.46 | 0.5 |  |
|  |  |  | | |  | | |  | |  | |  | | |  | |  | | |  | |  | **BDNF.co** | | -0.17 | | -0.08 | | | **-0.5** | -0.29 |  |
|  |  |  | | |  | | |  | |  | |  | | |  | |  | | |  | |  | |  | | **GABA.co** | **0.91** | | | **0.54** | **0.47** |  |
|  |  |  | | |  | | |  | |  | |  | | |  | |  | | |  | |  | |  | |  | **GLU.co** | | | 0.43 | **0.43** |  |
|  |  |  | | |  | | |  | |  | |  | | |  | |  | | |  | |  | |  | | | |  | **NO** | | **0.67** |  |
|  |  |  | | |  | | |  | |  | |  | | |  | |  | | |  | |  | |  | | | |  |  | | **TNF** |  |

Bold values indicate significant correlation.
