# Supplementary material for: Combined benznidazole and pentoxifylline therapy improves behavioral and cognitive changes in association with the regulation of systemic inflammatory profile in chronic experimental Chagas disease
Source: PLoS One. 2025 Nov 14;20(11):e0334708. doi: 10.1371/journal.pone.0334708 (PMC12617855; doi:10.1371/journal.pone.0334708)
Supplement: S7 Table — (DOCX) [file pone.0334708.s015.docx]

**Table S7. P-values of observed correlations between behavioral, cognitive and neurochemical variables.**

|  | **MBT** | **TST** | **EPMT.Entries** | **EPMT.Time** | | **H.memory** | | **NORT** | **ASET1** | | **ASET2** | | **TBARS.co** | | **TBARS.h** | | | **BDNF.co** | | | **GABA.co** | **GLU.co** | **NO** | | **TNF** |
| --- | --- | --- | --- | --- | --- | --- | --- | --- | --- | --- | --- | --- | --- | --- | --- | --- | --- | --- | --- | --- | --- | --- | --- | --- | --- |
| ***T. cruzi*** | **0.01** | 0.07 | **0.04** | **0.03** | | 0.30 | | 0.54 | 0.28 | | 0.39 | | **0.01** | | **0.01** | | | 0.31 | | 0.14 | | 0.41 | **<0.01** | | 0.44 |
|  | **MBT** | **<0.01** | **0.02** | **<0.01** | | **0.02** | | 0.86 | **0.01** | | **0.02** | | **<0.01** | | **<0.01** | | | 0.09 | | **<0.01** | | **0.01** | **0.03** | | 0.28 |
|  |  | **TST** | 0.12 | **0.01** | | 0.10 | | 0.12 | **0.02** | | **0.01** | | **0.02** | | **0.03** | | | **0.01** | | **0.02** | | 0.09 | **0.01** | | 0.14 |
|  |  |  | **EPMT.Entries** | 0.07 | | 0.55 | | 0.66 | 0.18 | | 0.11 | | **<0.01** | | **0.01** | | | **<0.01** | | 0.12 | | 0.30 | **0.02** | | 0.14 |
|  |  |  |  | **EPMT.Time** | | **0.02** | | 0.55 | 0.41 | | **0.02** | | **<0.01** | | **0.03** | | | 0.12 | | **<0.01** | | **0.04** | **0.04** | | 0.16 |
|  |  |  |  | |  | **H.memory** | | 0.59 | 0.69 | | **0.03** | | 0.41 | | 0.29 | | | 0.46 | | 0.34 | | 0.62 | 0.91 | | 0.81 |
|  |  |  |  | |  |  | | **NORT** | 0.97 | | 0.43 | | 0.26 | | 0.67 | | | 0.37 | | 0.49 | | 0.40 | 0.10 | | 0.13 |
|  |  |  |  | |  | |  |  | **ASET1** | | 0.36 | | 0.34 | | 0.33 | | | 0.14 | | **0.04** | | **0.04** | 0.38 | | 0.58 |
|  |  |  |  | |  | |  |  |  | | **ASET2** | | 0.13 | | 0.05 | | | 0.36 | | 0.10 | | 0.34 | 0.49 | | 0.39 |
|  |  |  |  | |  | |  |  | |  | |  | **TBARS.co** | | **<0.01** | | | 0.09 | | **<0.01** | | **0.02** | **<0.01** | | 0.06 |
|  |  |  |  | |  | |  |  | |  | |  |  | | **TBARS.h** | | | 0.14 | | 0.07 | | 0.33 | 0.09 | | 0.30 |
|  |  |  |  | |  | |  |  | |  | |  | |  | |  | **BDNF.co** | | | 0.33 | | 0.50 | **0.04** | | 0.38 |
|  |  |  |  | |  | |  |  | |  | |  | |  | |  | | |  | | **GABA.co** | **<0.01** | **0.04** | | **0.04** |
|  |  |  |  | |  | |  |  | |  | |  | |  | |  | | |  | |  | **GLU.co** | 0.09 | | **0.04** |
|  |  |  |  | |  | |  |  | |  | |  | |  | |  | | |  | |  |  | | **NO** | **0.02** |

Bold values indicate significant P-values.
